# The effectiveness of live music in reducing anxiety and depression among patients undergoing haemodialysis. A randomised controlled pilot study

**DOI:** 10.1371/journal.pone.0307661

**Published:** 2024-08-26

**Authors:** Miriam Serrano Soliva, Conrado Carrascosa López, Inmaculada Rico Salvador, Rafael Ortiz Ramón, Javier Villalón Coca, Rafael García Maset, Alicia García Testal

**Affiliations:** 1 Universitat Politècnica de València, Valencia, Spain, Conservatorio Profesional de Música de Buñol, Valencia, Spain; 2 Universitat Politècnica de Valencia, Valencia, Spain; 3 Nephrology Service at the Hospital de Manises, Valencia, Spain; 4 Data Analytics Department at the Hospital de Manises, Valencia, Spain, Universidad Internacional de Valencia–VIU, Valencia, Spain; 5 Healthcare Management, Vithassanidad, Valencia, Spain; University Hospital Cologne: Uniklinik Koln, GERMANY

## Abstract

**Background:**

Anxiety and depression are highly prevalent disorders among individuals undergoing chronic haemodialysis. For patients with kidney disease, the haemodialysis process often exacerbates these conditions. This study aims to investigate the effects of listening to live classical music on anxiety and depression scales during haemodialysis sessions.

**Methods:**

A randomised clinical trial was conducted with a group of patients who listened to live classical music during haemodialysis sessions, while the control group received treatment as usual. Anxiety and depression levels were assessed at baseline and after 4 weeks of listening to live music. The study comprised 90 patients.

**Results:**

The results demonstrated a significant decrease in anxiety and depression among the intervention group, who listened to music, compared to the control group, who did not receive this intervention. Specifically, the intervention group, presented a decrease in score on the anxiety scale of -5.35 (p < 0.001) points on average and a decrease in score on the depression scale of -5.88 (p < 0.001) points on average, while in the control group the levels worsened with the progression of time.

**Conclusion:**

It is concluded that listening to live classical music during haemodialysis sessions reduces anxiety and depression levels in HD patients. This conclusion adds value to listening to live music in the hospital context, specifically in this case, in haemodialysis rooms.

## Introduction

People tend to be more susceptible to experiencing anxiety and depression when faced with life-threatening circumstances, such as illnesses, surgical procedures, or aggressive medical treatments, such as haemodialysis.

Individuals suffering from chronic kidney disease (CKD) are commonly identified with anxiety and depression, essential components of negative affectivity [**1–4]**.

In many cases, the disorder is diagnosed after undergoing the changes involved in starting chronic treatment with haemodialysis (HD). Haemodialysis treatment involves changing lifestyles, schedules, some habits and even diet. They have to enter a new routine in which medical admissions, medication and dependence on a machine become the central elements of their life.

The emotional state produced by anxiety (fear, worry, stress) and the multiple disorders associated with depression, such as sadness, loss of interest, low self-esteem, feelings of guilt, loss of energy, disturbance of sleep and appetite, among others, can become chronic in these patients. These circumstances diminish their capacity to attend to their daily tasks and responsibilities [5].

The *National Institute for Health and Care Excellence* openly recognises the notoriety of depression in CKD [6]. The studies carried out by Kimmel [7] and Ibañez [8] report how anxiety and depression are more ingrained in patients when it is linked with another disease. They are, therefore, more persistent and resistant to treatment in CKD patients on HD than in the general population [9].

It is worth noting the study by Villaseca et al. [10], who analysed the prevalence of anxiety and depression in CKD patients on HD. They concluded that there is a high prevalence of anxiety and depression in this population.

It is well-known that music is an excellent tool, and if well-administered, it can significantly help reduce anxiety, depression and pain. Authors like Silva [11] or Harney [12] show that it can reduce the prevalence of anxiety. The study “An exploration of music. Listening in chronic pain" [13] notes it might help with distraction, relaxation and the feeling of control. In the same way Tang et al. [14] exhibit a more substantial effect in reducing depressive symptoms with music. In their comprehensive literature review, the music therapist Rodríguez-Rodríguez et al. [15] explores the significance of music therapy in paediatric oncology. The authors emphasize the occurrence of stressful situations among these patients, which induce emotional states in children, leading to psychological discomforts such as anxiety and depression. Along the same lines, the article written by Nguyen et al. [16] reports that Implementing music interventions combined with progressive muscle relaxation is feasible and had a trend in reducing anxiety, depression and stress levels for women with cancer.

Patients on haemodialysis suffer similar situations. Some benefits of music have been shown to reduce the degree of anxiety, stress and depression in these patients. The benefits of music on the quality of life have also been displayed in these studies [8, 17].

We highlight the article "Music Therapy during Haemodialysis: A Literature Review" [18], it brings together the latest contributions of music therapy concerning HD. It reveals that music therapy can promote time optimisation and stress reduction.

### Purpose and hypothesis

After a thorough analysis of all this background, we wonder whether listening to live classical music in haemodialysis rooms can reduce the degree of anxiety and depression of the patients.

Hence, our principal aim was to investigate the impact of live classical music exposure during haemodialysis sessions on the anxiety and depression levels of patients who receive it.

The effect of music on the quality of life of haemodialysis patients was also tested, and the results for quality of life were reported elsewhere [17].

## Material and methods

### Design

This is a pilot study with randomised intervention-control groups conducted over one month at the haemodialysis unit of the Nephrology Service at Hospital de Manises (Valencia). The trial study protocol (S2 File) was approved by the Research Ethics Committee and the Research Commission of the Instituto de Investigación Sanitaria La Fe, in Valencia (Spain), with registration number: 2018/0526, and in its procedures, complies with the GCP standards (CPMP/lCH/135/95 (S3 File), having obtained the prior written informed consent of the study subjects and in conformance with the Helsinki Declaration. The trial is registered retrospectively (for reasons unrelated to the researchers) on the platform https://clinicaltrials.gov/. Identificatory: NCT05729997.

The present study followed the CONSORT 2010 guidelines for conducting randomised clinical trials, and the CONSORT Checklist (S1 File) was completed [19, 20]. The intervention began on March 4, 2019, and continued until March 31, 2019.

All relevant data for this study can be found in the S5 File.

### Participants

Patient recruitment began two months prior to the musical intervention. Initially, all patients in the haemodialysis unit are provided with oral and written information regarding the study, including its risks, benefits, and the inclusion and exclusion criteria for participation in the research project.

Inclusion criteria: Patients aged 18 and above, capable of providing informed consent, and enrolled in the chronic haemodialysis program for more than 3 months. Exclusion criteria: Inability to listen to music, inability to respond to surveys, refusal to provide informed consent, age under 18, hospitalisation for more than 4 weeks, or hospitalisation within the last two weeks of the intervention. Patients who met the criteria for participation in the study were briefed on the objectives. Those who agreed to participate signed a consent (S4 File) form to enrol in the study.

### Randomisation

Patients were divided into two groups: the intervention group (IG), who listened to live music during their haemodialysis sessions, and the control group (CG), who did not receive the intervention. Since patients undergo dialysis in shared rooms, individualising the intervention of listening to live music was not feasible. Dialysis sessions were scheduled in fixed shifts: Monday, Wednesday, and Friday mornings (MWF-1); Monday, Wednesday, and Friday afternoons (MWF-2); Tuesday, Thursday, and Saturday mornings (TRS-1); or Tuesday, Thursday, and Saturday afternoons (TRS-2). Therefore, randomisation was conducted via a coin toss, and entire groups were assigned either to the control or intervention group. The randomisation and assignment of participants to each group (intervention or control) were conducted by research staff not involved in group evaluations, mitigating potential bias. The coin toss performed after all participants were recruited.

Randomisation assigned the intervention group (IG), who listened to live music during haemodialysis sessions, to the shifts (MWF-1) and (TRS-2), while the control group (CG), who did not receive the intervention, were assigned to the shifts (MWF-2) and (TRS-1). The intervention for the IG involved listening to live music performed by professional musicians in the haemodialysis room twice a week for four weeks.

Since it was impossible to conceal the intervention from patients, evaluators administering questionnaires were blinded to group assignment, minimising subjective biases, to enhance study rigour and scientific quality. Being blind prevented influencing the patient’s answers if the patient requested help to understand a question better.

### Sample size

The sample size was estimated considering an equal number of subjects in each of the four clusters: MWF-1 (IG), TRS-2 (IG), MWF-2 (CG), and TRS-1 (CG). Initially, a calculation was performed assuming randomisation at the patient level. To achieve statistical significance with an alpha risk of 0.05 and a beta risk of 0.2 (power of 80%) in a two-sided test, 72 subjects (36 subjects per arm) were deemed necessary to detect a difference of 2 points or more on the HAD scale. This estimation assumed a common standard deviation of 3, a correlation coefficient of 0.6 between initial and final measurements, and anticipated a dropout rate of 20%. The calculation was conducted using the sample size calculator GRANMO (version 8.0) based on the publication by Marrugat et al. [21].

Afterwards, this sample size was inflated by multiplying by the design effect in order to consider cluster design. The design effect formula, which assumes an equal number of patients per cluster, is 1+[(Nc-1)×ICC], where Nc is the number of participants per cluster, and ICC is the estimated intracluster correlation coefficient [22]. Considering four clusters (four haemodialysis sessions) and an ICC = 0.01, the design effect is 1+[(72/4-1)×0.01] = 1.17 and consequently the sample size was increased to 85.

### Music intervention

The musical element was administered in a live format rather than through recorded means, with each session extending for durations of 30 to 45 minutes. There were four sessions per week for 1month (twice for each IG: (MWF-1) and (TRS-2)). All the musicians were professionals. They performed live in the HD unit and always respected the space where doctors and nurses worked without interrupting them. One detail to highlight was that the musicians were placed in different locations inside the room so patients could see them and not only listen to them.

As many musicians were willing to participate in the study, it was possible to form and distribute various ensembles throughout the month. This distribution allowed patients to listen to distinct chamber ensembles and an extensive music repertoire from different periods and musical styles. Renaissance, baroque and classic pieces were played, as were pieces adapted for our formations (classic music formations) of songs by national and international artists, such as Joan Manuel Serrat, Ed Sheeran, and Paul McCartney, among others.

Distribution of groups and performed pieces:

***Solo oboe*:** "The Swan" by C. Saint-Saens, “Pavane” by G. Fauré, “Adagio” by Albinoni, “Fantasia for Solo Oboe No. 1” by Telemann and “Warrior Dances from Prince Igor” by A. Borodin.

***Piano and oboe*:** "Piensa en mi" by A. Lara, "Flower Duet" by Delibes, "Bohemian Rhapsody" by F. Mercury, "Habanera de Carmen" by Bizet, "Cinema Paradiso" by E. Morricone, "Cantata BWV 7 SichubenimLieben" by Bach, "What a Wonderful World" by L. Armstrong, "Ave Maria" by Schubert and "Gabriel’s Oboe" by E. Morricone,

***Cello and Oboe*:** "Cinema Paradiso" by E. Morricone, "Strangers in the Night" by F. Sinatra, "Moon River" by H. Mancini, "El Día que me quieras" by C. Gardel. "Lara’s Theme" by

M. Jarre,"La Senda del Tiempo" by CeltasCortos and "The Sound of Silence" by Simon and Garfunkel.

***Viola and cello*:** "Ave Maria" by Schubert, "Prelude 1st Suite for Solo Cello" by Bach, "Música Nocturna de Madrid" by Boccherini, "Perfect" by Ed. Sheeran and "La Dona e Mobile" by G. Verdi.

***Recorder and cello*:** "Largo from Xerxes" by Handel, four 16th century dances: "Ungaresca" by Mainerio; "Allemanda" by J.H.Schein; "Fuggerintanz" by Newsidler, "Pezzo Tedesco" (anonymous), "Yesterday" by J. Lennon and P. McCartney, "Oblivion" by A. Piazzolla and "Imagine" by J. Lennon.

***Violin*, *Cello and Piano*:** "La Valsd’Amélie" by Yann Tiersen, "El Día que me Quieras" by C. Gardel, "Irlandesa" by Bolling, " Life" by Ludovico, "Barcarolle" by J. Offenbach and "My Way" by F. Sinatra.

***Horn Trio*:** "We are the Champions" by F. Mercury for Queen, "Polovtsian Dance" by A. Borodin, "In the Hall of the Mountain King from Peer Gynt" by E. Grieg, "Pavana" by G. Fauré and "Jupiter" by Holst.

***Violin*, *Guitar and Voice*:** Kirtan music, which is said to be able to silence the mind, was performed. It is Indian spiritual chanting and traditional music.

***Clarinet quartet*:** "Sombrero de Tres Picos" by M. Falla, "Pasodoble Gallito" by S. Lope, "Argonaise de Carmen" by Bizet", "La Boda de Luis Alonso" by GerónimoGiménez, "Star Wars" by John Williams, "Czardas" by Monti and "Pasodoble Cielo Andaluz" by P. Marquina.

***Flute quartet*:** Beethoven’s "Septimino", Bach’s "Air", "Greensleeves", a traditional English folk melody, Mozart’s "Little Night Serenade", and "Tea for Two" by Vincent Youmans.

A decision was made to end all performances with the theme of the film "*Life is Beautiful"* by Nicola Piovani so that patients, doctors and other healthcare staff could relate to and identify the piece as the end of performances.

These repertoire pieces were programmed to be as familiar and catchy as possible for patients. Musicians often improvised to interpret personal requests.

### Outcome variables

Anxiety and depression levels were recorded at the baseline, before starting music interventions, and at the end after the last music session.

Anxiety and depression levels were measured with the Hospital Anxiety and Depression Scale (HAD) [23] questionnaire, which is widely used to detect emotional distress (anxiety and depression) in different diseases [24, 25].

It is a questionnaire composed of 14 questions divided into two subsections, one for the Anxiety scale (HAD-A) with seven items and the other for the Depression scale (HAD-D) with seven more items. The evaluators distributed the questionnaires to the patients during the haemodialysis session so they could fill them out themselves, as long as the haemodialysis was proceeding stably and without complications.

However, the evaluators who distributed the questionnaires were unaware of which group the patients belonged to.

### Other variables register

In addition to anxiety and depression levels, some descriptive variables were included in the analysis to understand each group’s characteristics better. Gender, age, vascular access (catheter or arteriovenous fistula) and time on treatment were recorded. Some additional medical data were recorded: whether they were on psychotropic drugs or analgesics, serum haemoglobin (gr/dl), serum albumin (gr/dl), Kt/V Daugirdas 2nd generation based on serum urea specifications before and after HD, and blood pressure (mmHg) on the day that surveys were conducted. Data were collected from the electronic medical record.

### Statistical analysis

A descriptive analysis was carried out in the first place to define the characteristics of each group and session. The dependent and independent variables were presented using the mean, standard deviation (SD), median, and the first and third quartiles (interquartile range, IQR) with continuous variables and using relative and absolute frequencies with categorical variables. The reason for this analysis was to visualise their distribution and look for possible error sources if outliers were present.

The variability between patient-level outcomes of individual sessions/clusters in this preliminary study was initially explored using non-parametric Mann-Whitney-Wilcoxon tests. We compared session/cluster results by assessing the difference between pre- and post-intervention scores for anxiety and depression as the outcome. Associations with p-values <0.05 were considered statistically significant. Additionally, Bonferroni correction was employed to control for multiple comparison issues when conducting multiple statistical tests on the same variable.

This analysis was performed using the difference between pre and post-intervention scores for anxiety and depression as the outcome.

The intracluster correlation of patient-level outcomes within clusters and groups was also assessed. According to CONSORT 2010 guidelines [26], when clustering is present in a randomised clinical trial, cluster or individual participant level result and a coefficient of intracluster correlation for each primary outcome must be reported. To this end, the intracluster correlation coefficient (ICC) is the most common statistic. The ICC provides a similarity measure by comparing the variance within clusters with the variance between clusters. Values of ICC range from 0 to 1: Small values for ICC imply that the within-cluster variance is much greater than the between-cluster variance, while larger values for ICC are related to a higher between-cluster variance [27]. This study calculated ICCs for pre-intervention, post-intervention and pre/post-intervention difference outcomes (anxiety and depression) using one-way nested random effects models [28]. Point estimates for the ICCs are accompanied by bootstrap 95% confidence intervals. Specifically, we resampled with replacement to produce 1000 bootstrap samples, calculated the ICC in each iteration and reported empirical 95% bootstrap confidence intervals [29].

The results of these preliminary tests may indicate that our hypothesis is valuable. However, causal inferences at the group level must be interpreted carefully in this case since our analyses do not consider the clustering effect. The small number of clusters per arm (two sessions per group) in our design restricts the use of more detailed analyses with adjustments for potential confounders, thus limiting the statistical precision of our study.

Employed statistical software:

The statistical analyses were conducted utilizing R software (version 3.4.1; R Foundation for Statistical Computing, Vienna, Austria) and employing the lme4 package (version 1.1–17).

## Results

### Descriptive analysis

Of the 120 patients in the HD unit where the study was conducted, 92 met the inclusion criteria. Of this, 90 were finally included because two were excluded for reasons unrelated to the study. No missing values were present in this study.

**Fig 1:**
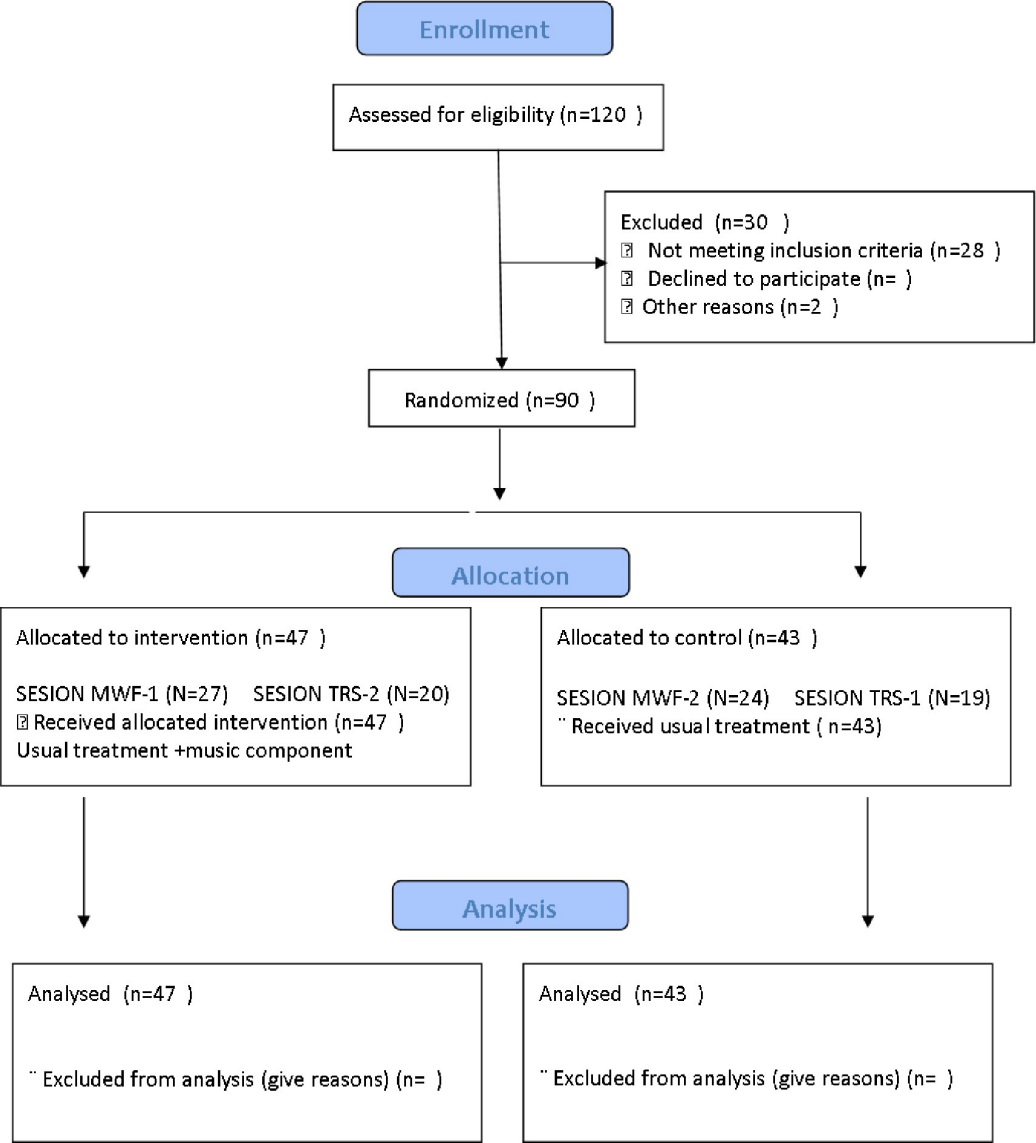
Flow diagram.

Randomisation assigned 47 patients to the intervention group (IG) belonging to the HD shift (MWF-1) (TRS-2) and 43 patients to the control group (CG), belonging to the HD shift (MWF-2) (TRS-1). Fig 1 shows the Flow Diagram with the details.

The descriptive analysis results showed each group’s characteristics in Table 1 below.

**Table 1:** Descriptive characteristics of the patients included in the study.

	IG (N = 47)		CG (N = 43)	
	Session MWF-1 (N = 27)	Session TRS-2 (N = 20)	Session MWF-2 (N = 24)	Session TRS-1 (N = 19)
Gender				
*Female n (%)*	13 (48.15%)	5 (25.00%)	11 (45.83%)	11 (57.89%)
*Male n (%)*	14 (51.85%)	15 (75.00%)	13 (54.17%)	8 (42.11%)
Age (years)				
*Mean (SD)*	70.41 (11.29)	77.75 (9.00)	73.12 (10.22)	79.21 (7.94)
*Median (IQR)*	73.00 (65.50, 78.50)	80.00 (75.00, 84.00)	75.00 (65.00, 81.25)	80.00 (77.00, 84.00)
Haemodialysis vintage (months)				
*Mean (SD)*	72.15 (60.52)	51.45 (36.95)	65.17 (43.59)	54.53 (38.05)
*Median (IQR)*	59.00 (29.50, 92.00)	42.50 (18.25, 75.00)	46.50 (33, 98.25)	51.00 (24.00, 79.00)
Vascular access kind				
*CVC n (%)*	5 (18.52%)	3 (15.00%)	5 (20.83%)	4 (21.05%)
*AVF n (%)*	22 (81.48%)	17 (85.00%)	19 (79.17%)	15 (78.95%)
Kt/V				
*Mean (SD)*	1.41 (0.27)	1.56 (0.48)	1.55 (0.19)	1.52 (0.17)
*Median (IQR)*	1.37 (1.24, 1.59)	1.42 (1.38, 1.58)	1.54 (1.45, 1.73)	1.54 (1.42, 1.61)
Haemoglobin				
*Mean (SD)*	11.21 (1.14)	11.46 (1.11)	11.57 (0.90)	11.54 (1.24)
*Median (IQR)*	11.20 (10.35, 12.1)	11.40 (10.85, 12.22)	11.45 (11.20, 12.10)	11.80 (10.65, 12.15)
Systolic blood pressure				
*Mean (SD)*	140.15 (22.29)	133.55 (16.59)	132.29 (22.81)	136.11 (34.93)
*Median (IQR)*	142.00 (124.50, 154.50)	134.5 (121.25, 145.75)	139.5 (123.00, 144.75)	132.00 (114.50, 157.50)
Diastolic blood pressure				
*Mean (SD)*	60.78 (12.73)	55.9 (11.07)	57.08 (12.88)	54.84 (11.64)
*Median (IQR)*	57.00 (52.50, 68.50)	56.50 (48.75, 62.00)	57.50 (48.50, 66.50)	58.00 (46.00, 62.00)
Albumin				
*Mean (SD)*	3.93 (0.35)	3.56 (0.44)	3.90 (0.31)	3.83 (0.53)
*Median (IQR)*	4.00 (3.95, 4.05)	3.60 (3.22, 3.85)	4.00 (3.80, 4.00)	4.00 (3.75, 4.05)

*IG (Intervention group), CG (Control group), MWF-1(Monday, Wednesday, and Friday mornings, MWF-2 (Monday, Wednesday, and Friday afternoons), TRS-1 (Tuesday, Thursday, and Saturday mornings), TRS-2 (Tuesday, Thursday, and Saturday afternoons)

### Exploratory analysis

As previously mentioned, the difference between pre and post-intervention scores for anxiety and depression of each patient was computed and evaluated in subsequent analyses.

The CG sessions (MWF-2 and TRS-1) showed an increase in anxiety and depression levels, worsened with the progression of time, as shown in Table 2. On the contrary, the sessions in the IG (MWF-1 and TRS-2) showed a severe negative difference between pre and post-intervention for both Anxiety and Depression scores, which means that the scales were reduced after intervention in these sessions.

**Table 2:** Time course of the anxiety and depression levels for the clusters included in the control group (CG) and the intervention group (IG).

Variable	Group	Session/Cluster	Pre-intervention*	Post-intervention*	Pre and post-intervention difference*
Anxiety	CG	MWF-2 (N = 24)	6.46 (5.18)	6.92 (5.04)	0.46 (1.14)
		TRS-1 (N = 19)	6.11 (3.6)	7.53 (4.38)	1.42 (3.24)
	IG	MWF-1 (N = 27)	7.63 (5.96)	2.59 (2.96)	-5.04 (4.58)
		TRS-2 (N = 20)	6.05 (3.95)	2.35 (2.54)	-3.70 (3.23)
Depression	CG	MWF-2 (N = 24)	7.25 (3.61)	8.25 (3.49)	1.00 (2.09)
		TRS-1 (N = 19)	8.26 (4.21)	9.00 (3.93)	0.74 (1.45)
	IG	MWF-1 (N = 27)	8.07 (5.29)	3.56 (3.37)	-4.52 (4.15)
		TRS-2 (N = 20)	8.85 (5.24)	3.20 (3.14)	-5.65 (4.00)

* Mean (SD)*IG (Intervention group), CG (Control group), MWF-1(Monday, Wednesday, and Friday mornings, MWF-2 (Monday, Wednesday, and Friday afternoons), TRS-1 (Tuesday, Thursday, and Saturday mornings), TRS-2 (Tuesday, Thursday, and Saturday afternoons)

**Fig 2:**
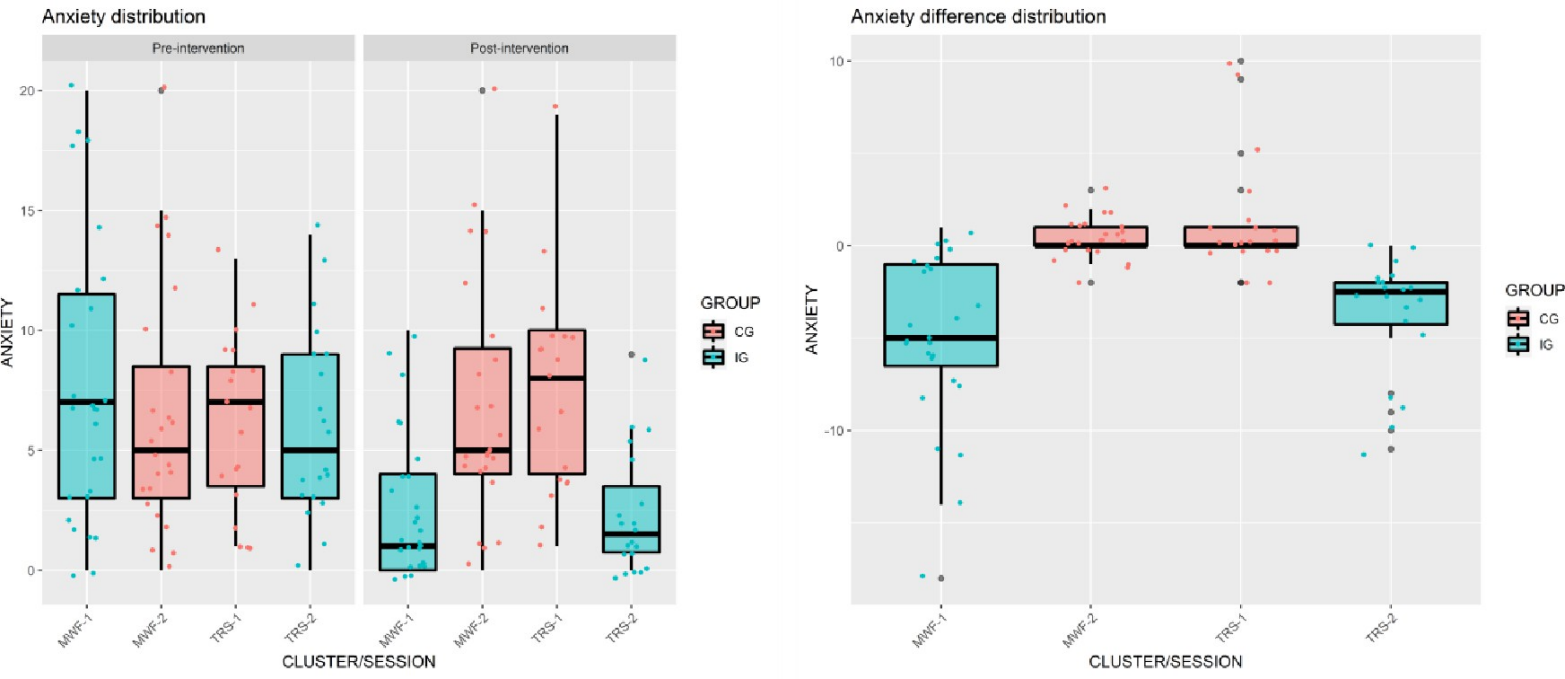
Distribution of pre- and post-intervention scores for anxiety.

**Fig 3:**
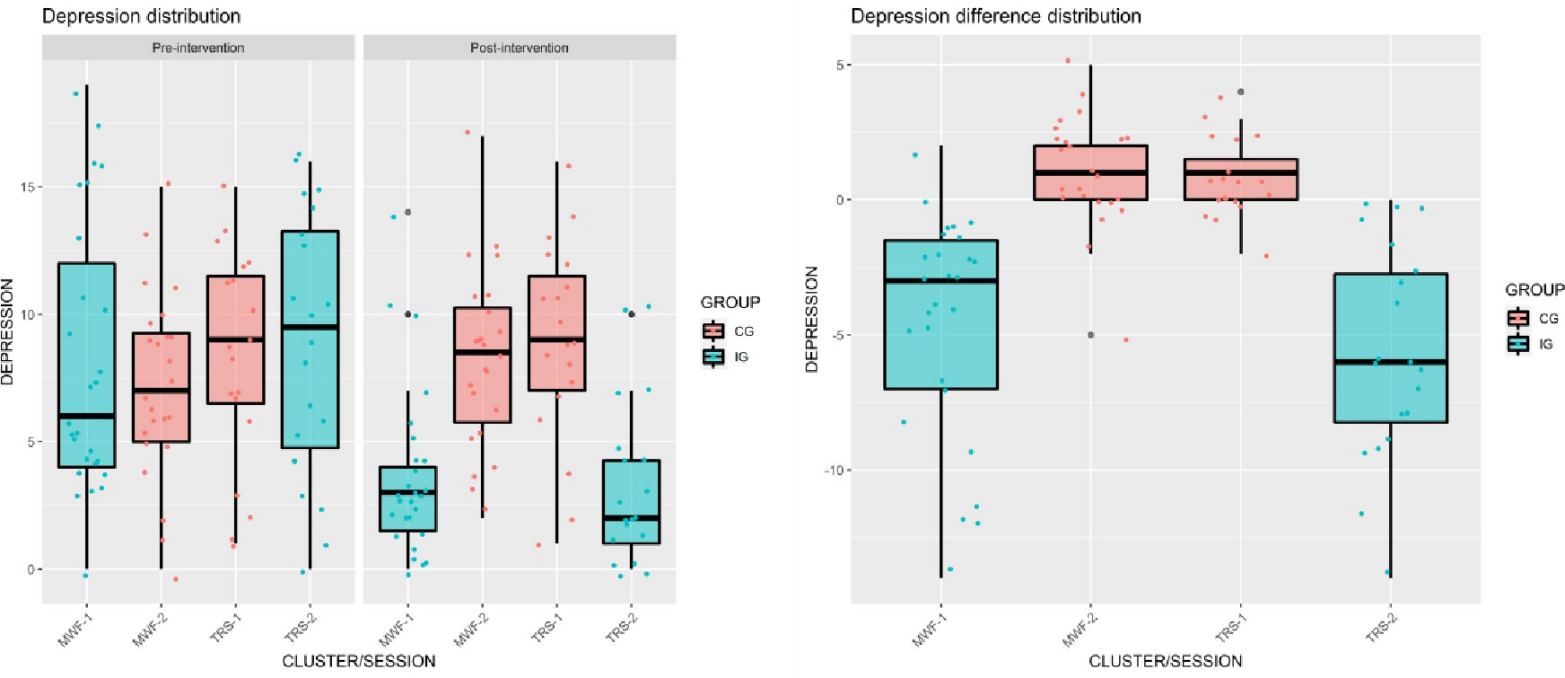
Distribution of pre- and post-intervention scores for depression.

The Mann-Whitney-Wilcoxon tests (Table 3) showed that differences between sessions/clusters of the same group were not significant in terms of anxiety and depression differences. In particular, the difference between the two sessions in CG presented a p = 0.788 for the anxiety difference scale and p = 0.483 for the depression difference scale, and the difference between the two sessions in IG presented a p = 0.387 for the Anxiety difference scale and p = 0.321 for the Depression difference scale. In contrast, comparisons between sessions of different groups were all significant in terms of both anxiety and depression differences even after Bonferroni correction (p < 0.05/6). Several box plots were also presented in Fig 2 for anxiety and in Fig 3 for depression in order to report these results graphically. As previously stated, causal inferences must be interpreted cautiously since our analyses do not consider the clustering effect.

**Table 3:** Mann-Whitney-Wilcoxon tests results for comparisons between pre and post-intervention differences of individual clusters.

Variable	Comparison	*p*-value
Anxiety difference	MWF-2 (CG) vs. TRS-1 (CG)	0.788
	MWF-1 (IG) vs. TRS-2 (IG)	0.387
	MWF-2 (CG) vs. MWF-1 (IG)	<0.001
	MWF-2 (CG) vs. TRS-2 (IG)	<0.001
	TRS-1 (CG) vs. MWF-1 (IG)	<0.001
	TRS-1 (CG) vs. TRS-2 (IG)	<0.001
	MWF-2+TRS-1 (CG) vs. MWF-1+TRS-2 (IG)	<0.001
Depression difference	MWF-2 (CG) vs. TRS-1 (CG)	0.483
	MWF-1 (IG) vs. TRS-2 (IG)	0.321
	MWF-2 (CG) vs. MWF-1 (IG)	<0.001
	MWF-2 (CG) vs. TRS-2 (IG)	<0.001
	TRS-1 (CG) vs. MWF-1 (IG)	<0.001
	TRS-1 (CG) vs. TRS-2 (IG)	<0.001
	MWF-2+TRS-1 (CG) vs. MWF-1+TRS-2 (IG)	<0.001

*IG (Intervention group), CG (Control group), MWF-1(Monday, Wednesday, and Friday mornings, MWF-2 (Monday, Wednesday, and Friday afternoons), TRS-1 (Tuesday, Thursday, and Saturday mornings), TRS-2 (Tuesday, Thursday, and Saturday afternoons)

The evaluation of the intracluster correlation of patient-level outcomes within clusters and groups revealed that ICC based on data in the post-intervention phase was much larger than ICC based on the pre-intervention data (Table 4). These results suggest that the between-cluster variance was insignificant at pre-intervention compared to the within-cluster variance (ICC ≤ 0.01) and that the intervention induced more variation between sessions and groups.

**Table 4:** ICCs (and 95% empirical bootstrap Cls) calculated for patient-level outcomes (anxiety and depression differences) within the four clusters and the two groups (control and intervention).

Variable	Clustering level	ICC (95% CI) pre-intervention	ICC (95% CI) post-intervention	ICC (95% CI) difference pre/post-intervention
Anxiety	Within 4 sessions	0.010 (-0.043–0.161)	0.313 (0.190–0.522)	0.461 (0.361–0.588)
	Within 2 groups	0.001 (-0.023–0.120)	0.423 (0.263–0.609)	0.555 (0.458–0.661)
Depression	Within 4 sessions	0.005 (-0.042–0.147)	0.413 (0.252–0.618)	0.524 (0.420–0.660)
	Within 2 groups	0.004 (-0.024–0.121)	0.524 (0.334–0.699)	0.624 (0.513–0.733)

We may note that some lower CIs of pre-intervention ICCs are negative. This occurs because the expression for calculating ICC [28] may estimate negative values, although they are generally expected to be positive. A negative ICC is simply an unfortunate estimate that may happen by chance, especially if the sample size is small. In such cases, the general practice is to set the negative value equal to zero [30].

## Discussion

The present study shows that live music during HD may reduce levels of anxiety and depression.

The power of music to soothe was discovered spontaneously and intuitively by primitive mothers who hummed to their babies to relax and calm their crying. There are also biblical passages that refer to the power of music, and even the Greeks recognised its benefits for distracting the sick. Pythagoras quoted: "Harmonic music has the power to provoke various emotions, to cure the ills of the spirit, the soul, the body and thus relieve people", which is why he called it "musical medicine". Its use was limited to diseases of the soul, emotional disorders and neurological diseases [31]. According to all these descriptions, anxiety and depression were probably the so-called "soul diseases" in those times and would have been treated with the "musical medicine" described by Pythagoras.

Our research shows that playing live music during treatment while CKD patients were receiving HD lowered levels of anxiety and depression.

In 2017, a study by Shabandokht-Zarmi et al. [32] measured the calming effect of music and pain reduction at the time of fistula puncture in HD patients by dividing patients into three groups: A (headphone music and listening to favourite songs); B (headphones without sound); C (no intervention). The results showed a difference in pain at the puncture time between groups A and C and between groups A and B, but there was no difference between groups B and C. The authors concluded that music had a calming effect and relieved pain upon fistula puncture.

The research carried out by Georgina AlcántaraAlencar Melo et al. [33] shares similarities with the present study because it evaluated the therapeutic effect of music on CKD patients’ anxiety. Those authors also compared an IG that listened to music to a CG, but unlike our study, patients listened to recorded music through headphones. However, the results align with ours because they showed lower anxiety levels in the IG, with levels remaining the same or worsening in the CG.

The novelty of our results compared to the reviewed literature lies in us not only analysing the parameter of anxiety level but also depression level. The result of comparing the CG and the IG showed that both groups evolved differently in terms of anxiety and depression between the two-time points, and this difference in evolution was positive for the IG.

Perhaps the most relevant novelty was to administer music to patients directly, live music, in the HD room, with musicians performing there rather than music played through electronic devices. The research team previously prepared musicians to place them in the participants’ casuistry, emotional state, and diseases. Furthermore, they could act according to any incident and were always guided and advised by the research team. They connected perfectly with patients, made them participate in their actions, and even frequently interpreted their requests.

Perhaps the fact that the patients could see the musicians and not just hear them could have had a positive effect on the patients since this social contact with the musicians could have enhanced the effect of music in reducing anxiety and depression levels.

Although the results seem to support the possibility that the effect of being in direct face-to-face contact with musicians was enhanced, we cannot state this for sure. It is merely an assumption that should be the subject of future research.

Another question we think is important to share to encourage research is the upbeat effect music could have on the musicians and the healthcare staff who carried out their tasks during the study and with whom spaces were shared. It is essential to encourage research on this topic.

The healthcare staff told us in casual conversations how they felt about the interventions. According to their opinions, they felt happier, friendlier and more active. The head nurse of the nephrology unit told us that his team seemed more animated and motivated. This effect was not recorded or quantified.

The musicians had similar comments. They expressed much satisfaction with seeing the great help that they contributed. They were grateful to play on a different stage from what they were used to. They also stated that it was much more gratifying for them to play to help, and they felt they were valuable as both professionals and people. In short, we firmly believe that music should be present much more in patients’ lives as part of their treatments. The convergence of music and medicine represents a potentially inherent remedy devoid of adverse effects. Perhaps the quintessential human attribute of music lies in its capacity to serve as a therapeutic instrument facilitating healing.

We are aware of the limitations of our research. First and foremost, this preliminary study includes only two clusters per arm, so our findings are difficult to use for causal inference about group differences. Further research with more sessions per group should be carried out in order to allow more sophisticated statistical analyses, such as linear mixed regression models adjusted for potential confounders that strengthen the statistical precision. Additionally, we should highlight that these results demonstrate a short-term effect. We do not know if the levels of distress will return to the baseline when they continue to undergo haemodialysis without music. Although a one-month intervention significantly improved patients, it would be interesting to re-evaluate them after a more extended intervention period. Future research could study the effect of live music on other chronic diseases that involve lengthy hospital stays, such as cancer patients and organ transplants. Randomised, blinded, multicenter clinical trials are needed to confirm the evidence of the effect of music on anxiety and depression in HD patients.

## Conclusion

In conclusion, listening to live music during haemodialysis sessionhelps reduce anxiety and depression levels in HD patients. It is necessary to investigate this topic further, as live music could be a valuable tool in treating other chronic patients. This conclusion also adds value to listening to live music in the hospital context, specifically and in this case, in haemodialysis rooms.

## Supporting information

S1 File CONSORT checklist. (ZIP)

S2 File Trial study protocol. (ZIP)

S3 File Ethics committee. (TIF)

S4 File Written consent. (TIF)

S5 File Supporting information data. (ZIP)
