# Supplementary figures and images for: The effectiveness of live music in reducing anxiety and depression among patients undergoing haemodialysis. A randomised controlled pilot study
Source: PLoS One. 2024 Aug 26;19(8):e0307661. doi: 10.1371/journal.pone.0307661 (PMC11346941; doi:10.1371/journal.pone.0307661)

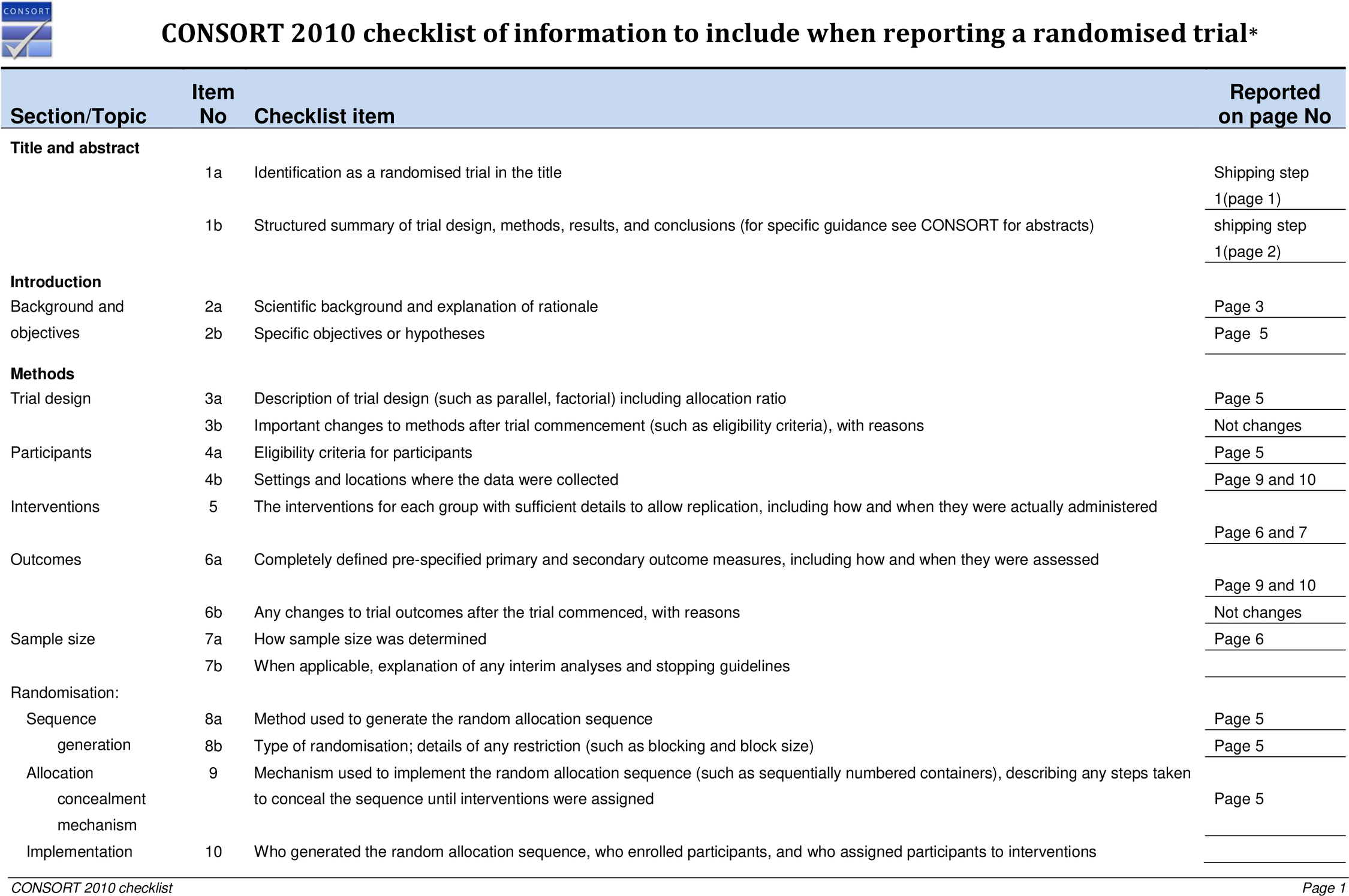

Supplement: S1 File — (ZIP) [file pone.0307661.s001.zip › S1 File.CONSORT 2010 Checklist (2).tif]

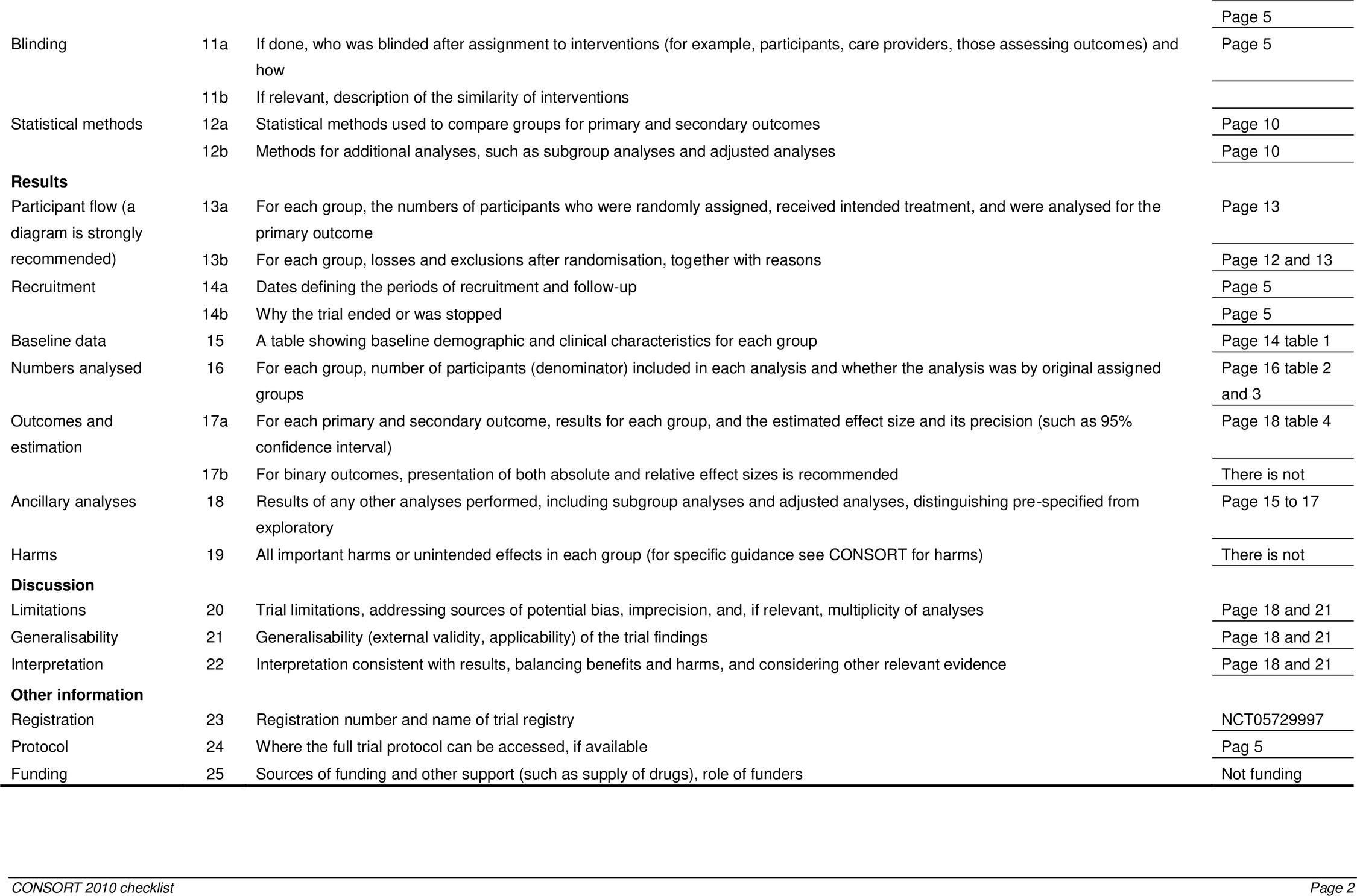

Supplement: S1 File — (ZIP) [file pone.0307661.s001.zip › S1 File.CONSORT 2010 Checklist (3).tif]

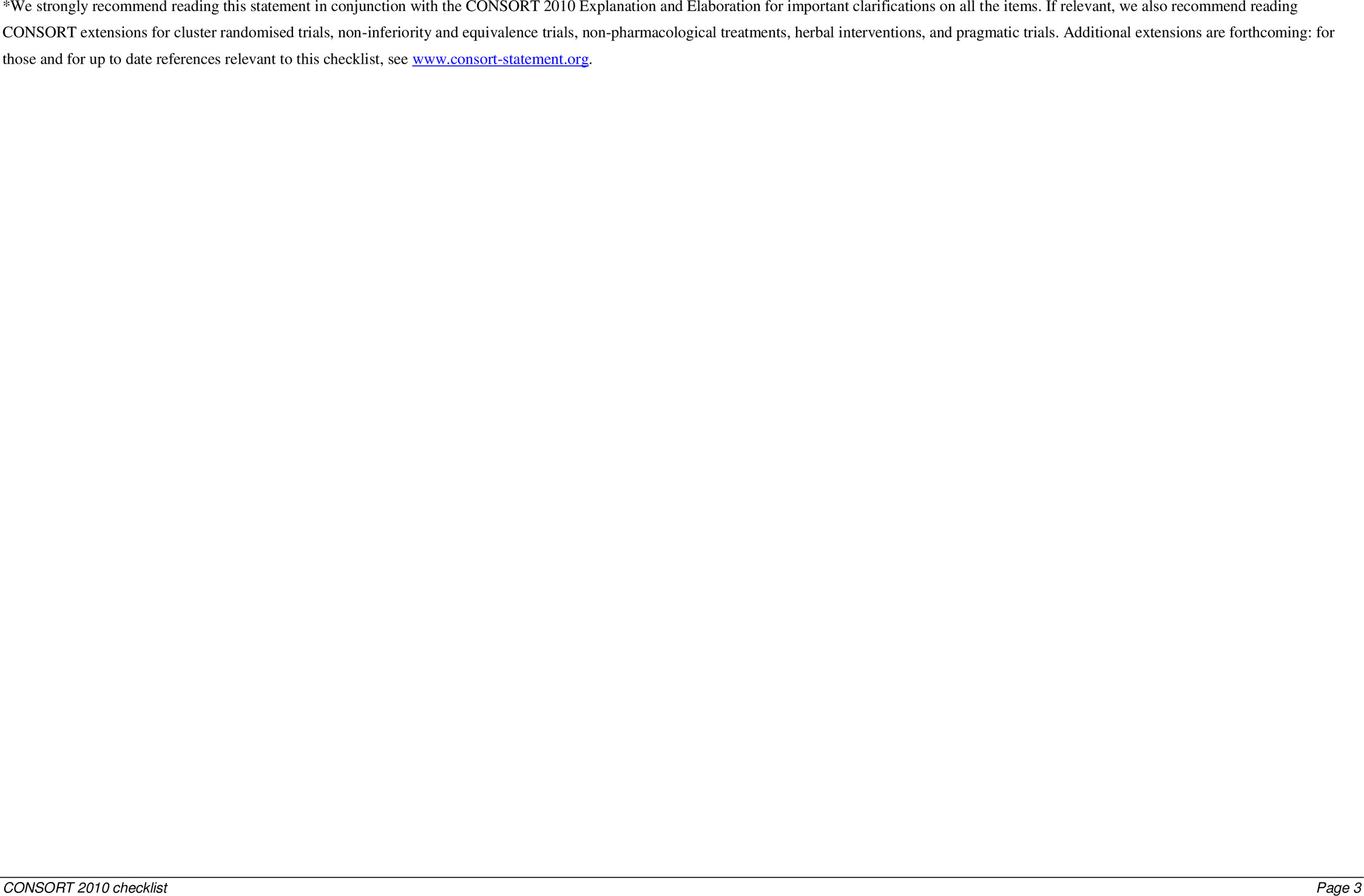

Supplement: S1 File — (ZIP) [file pone.0307661.s001.zip › S1 File.CONSORT 2010 Checklist.tif]

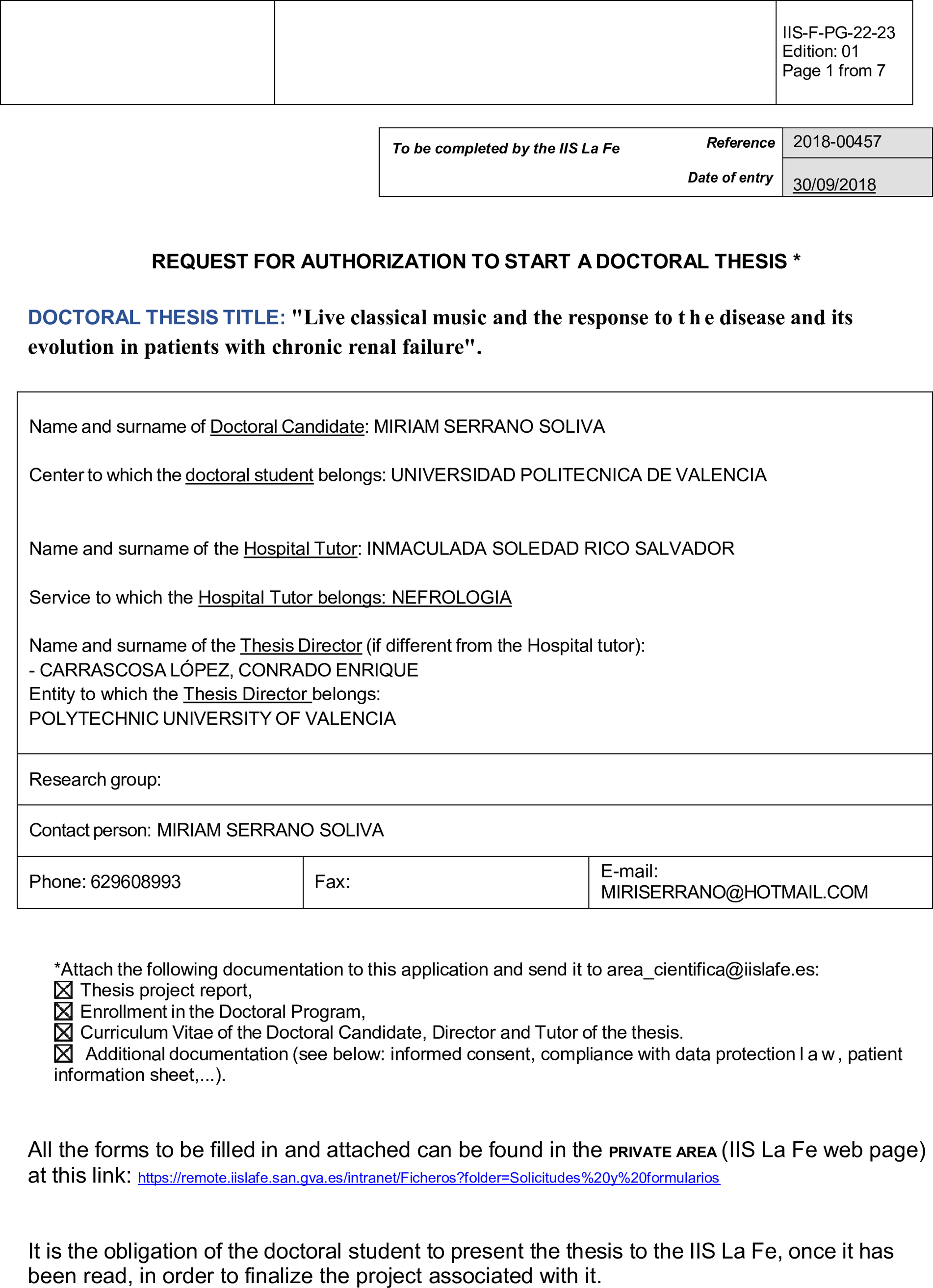

Supplement: S2 File — (ZIP) [file pone.0307661.s002.zip › S2 File.Trial study protocol (2).tif]

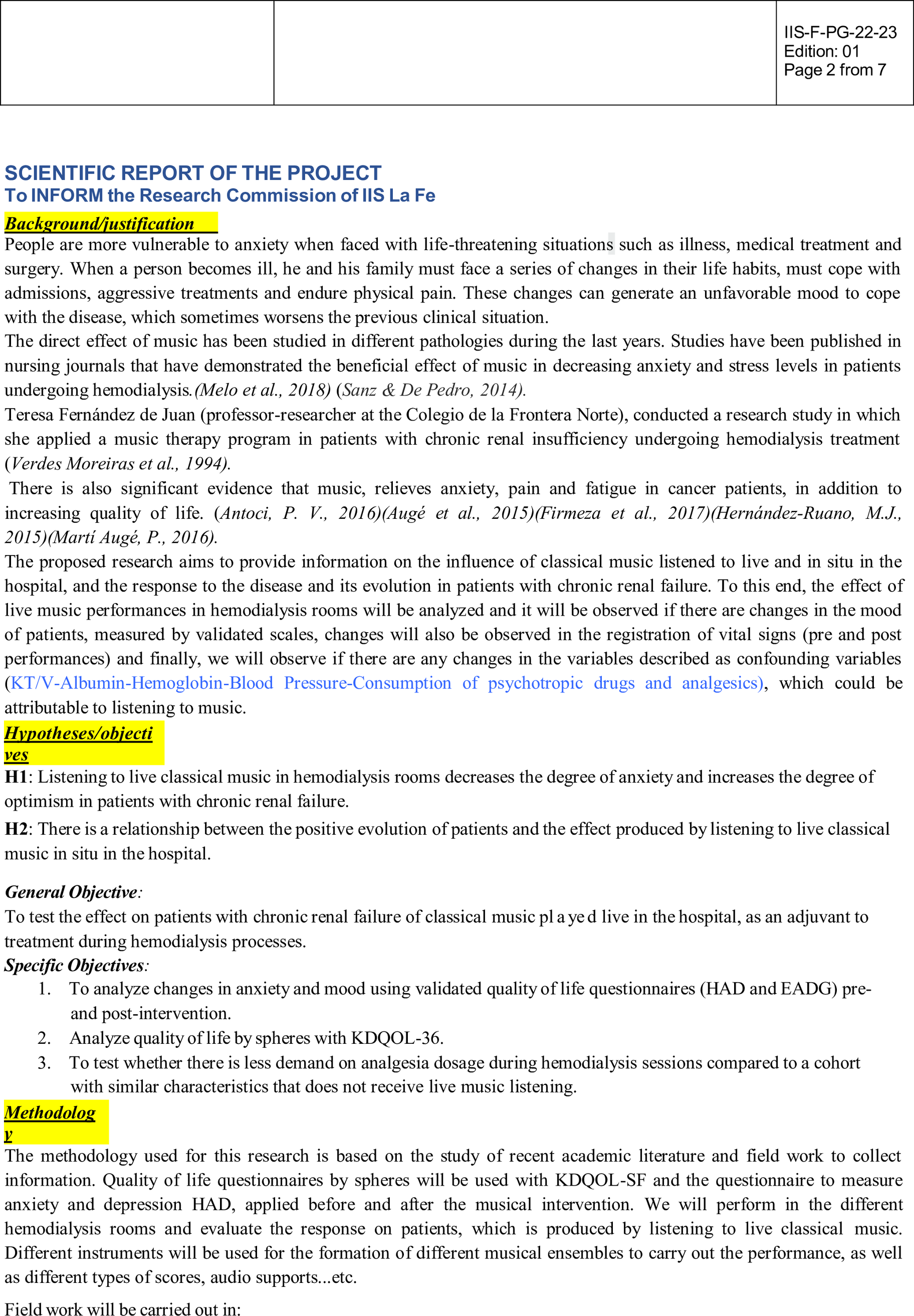

Supplement: S2 File — (ZIP) [file pone.0307661.s002.zip › S2 File.Trial study protocol (3).tif]

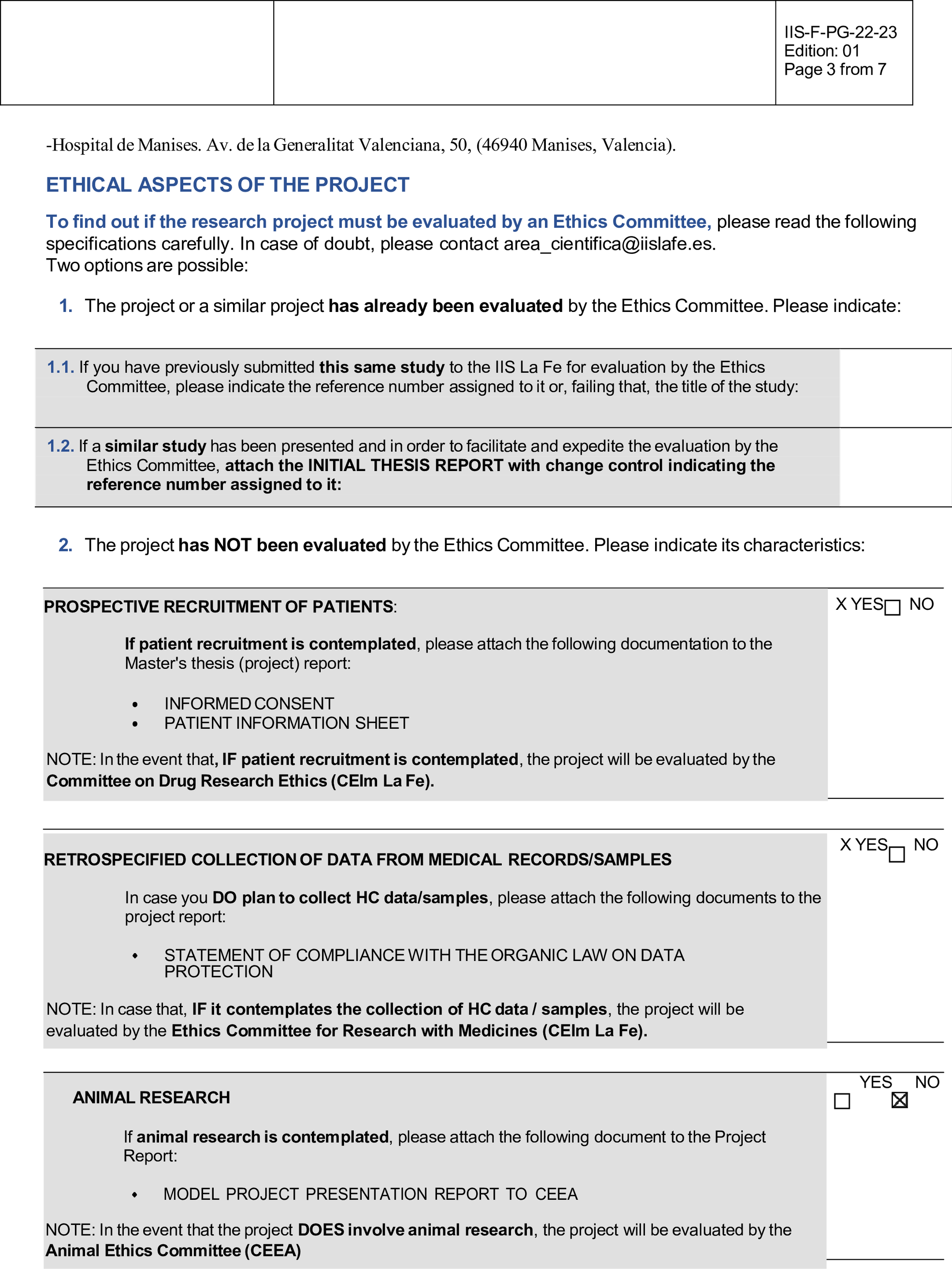

Supplement: S2 File — (ZIP) [file pone.0307661.s002.zip › S2 File.Trial study protocol (4).tif]

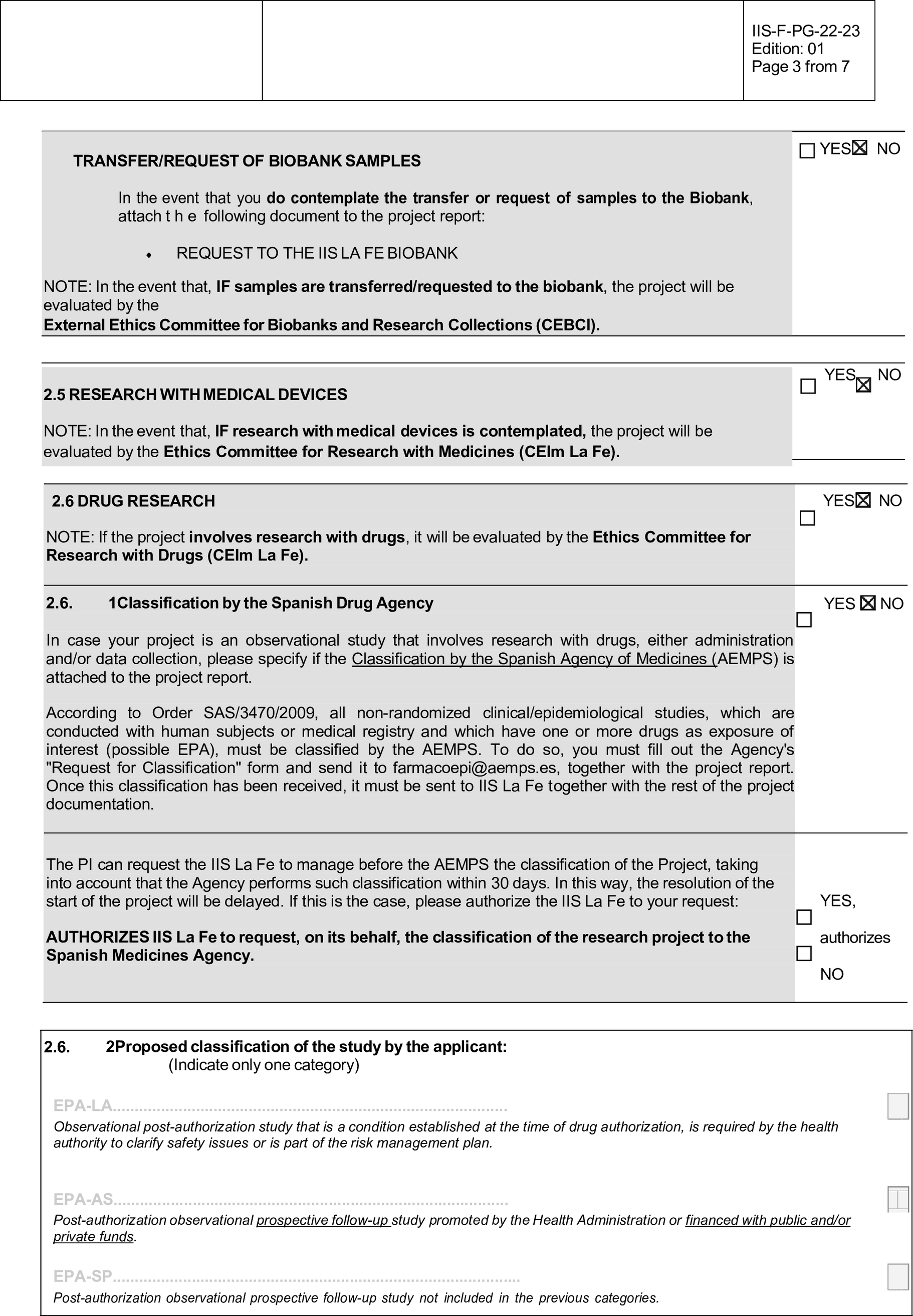

Supplement: S2 File — (ZIP) [file pone.0307661.s002.zip › S2 File.Trial study protocol (5).tif]

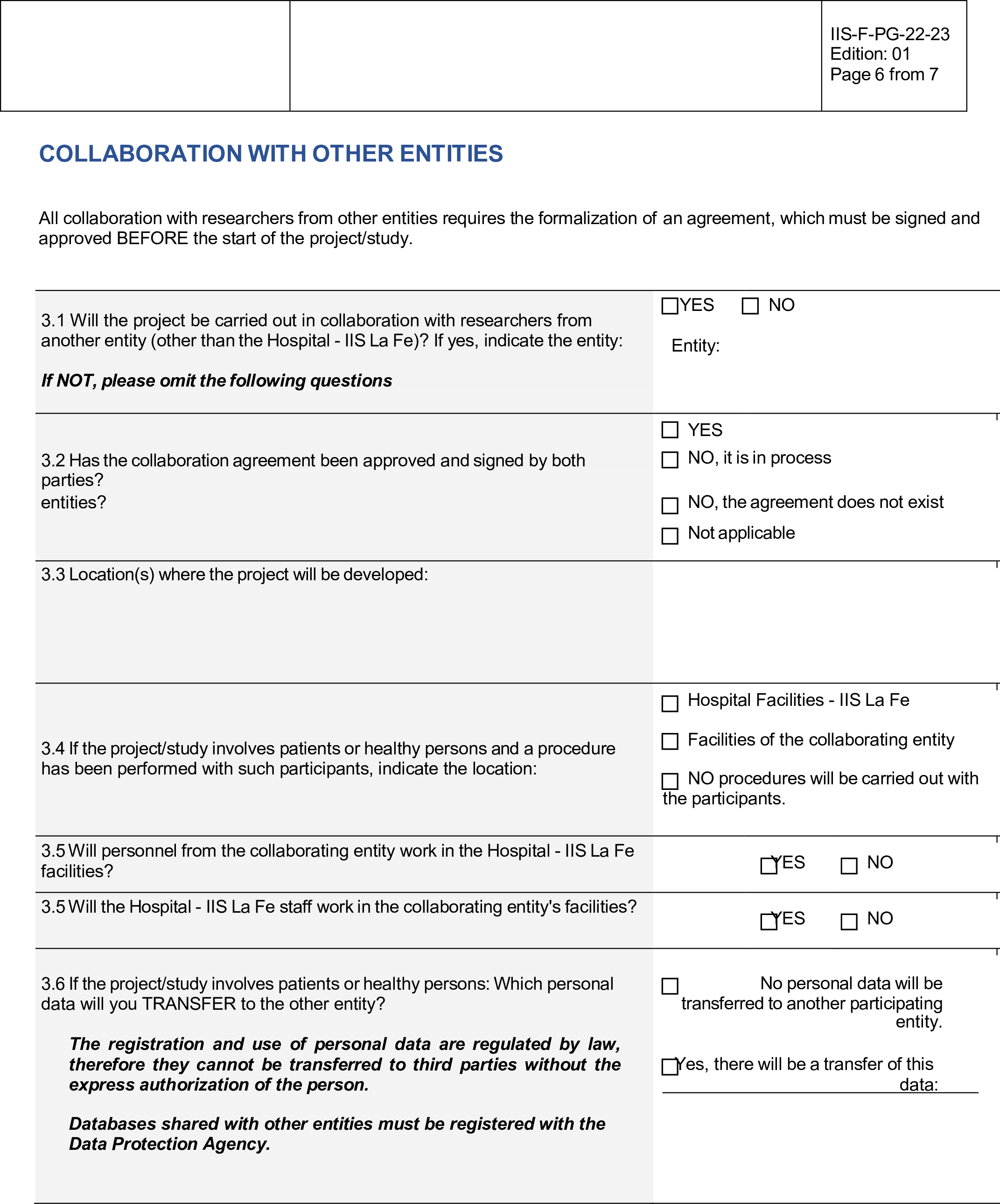

Supplement: S2 File — (ZIP) [file pone.0307661.s002.zip › S2 File.Trial study protocol (6).tif]

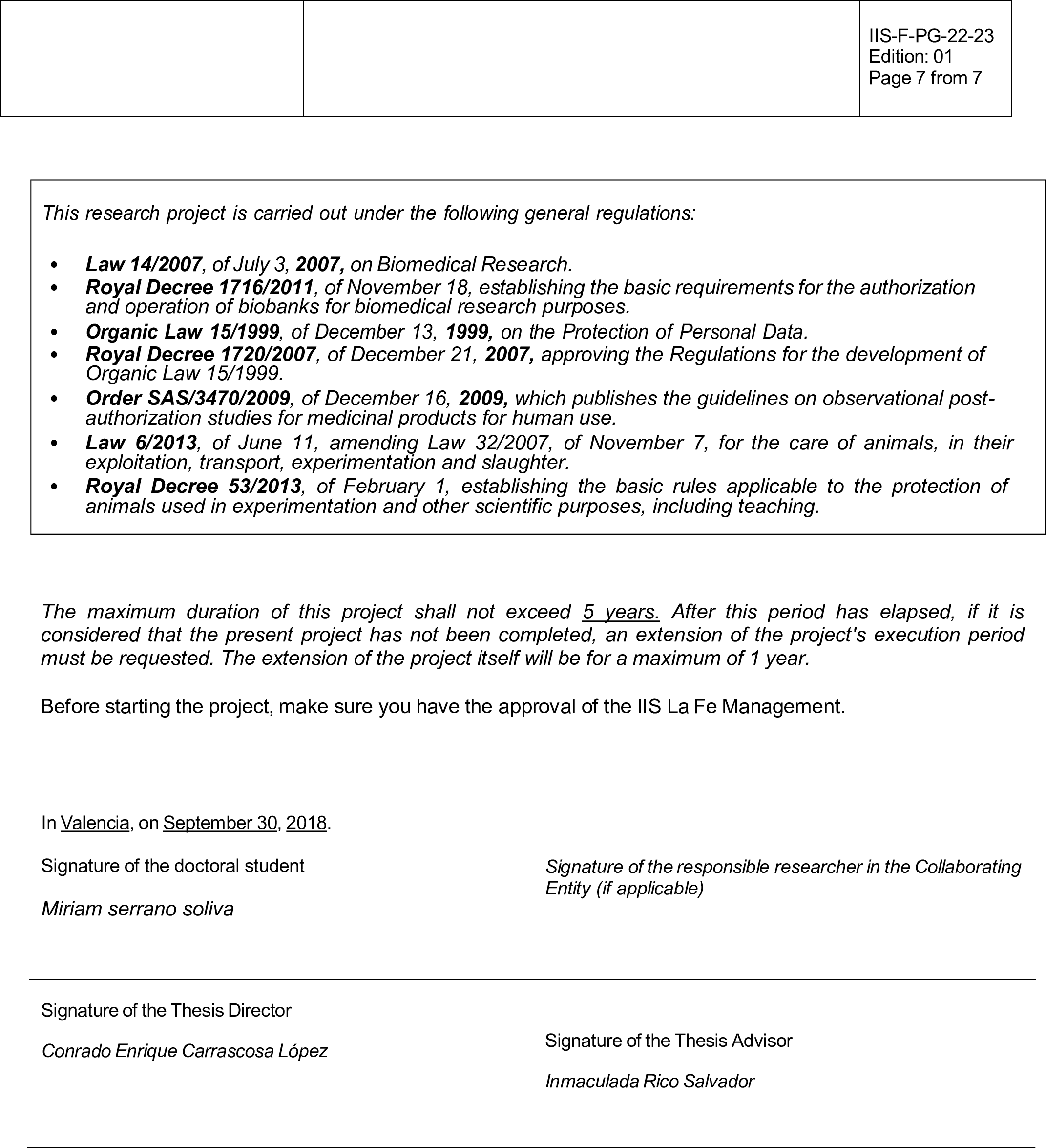

Supplement: S2 File — (ZIP) [file pone.0307661.s002.zip › S2 File.Trial study protocol.tif]

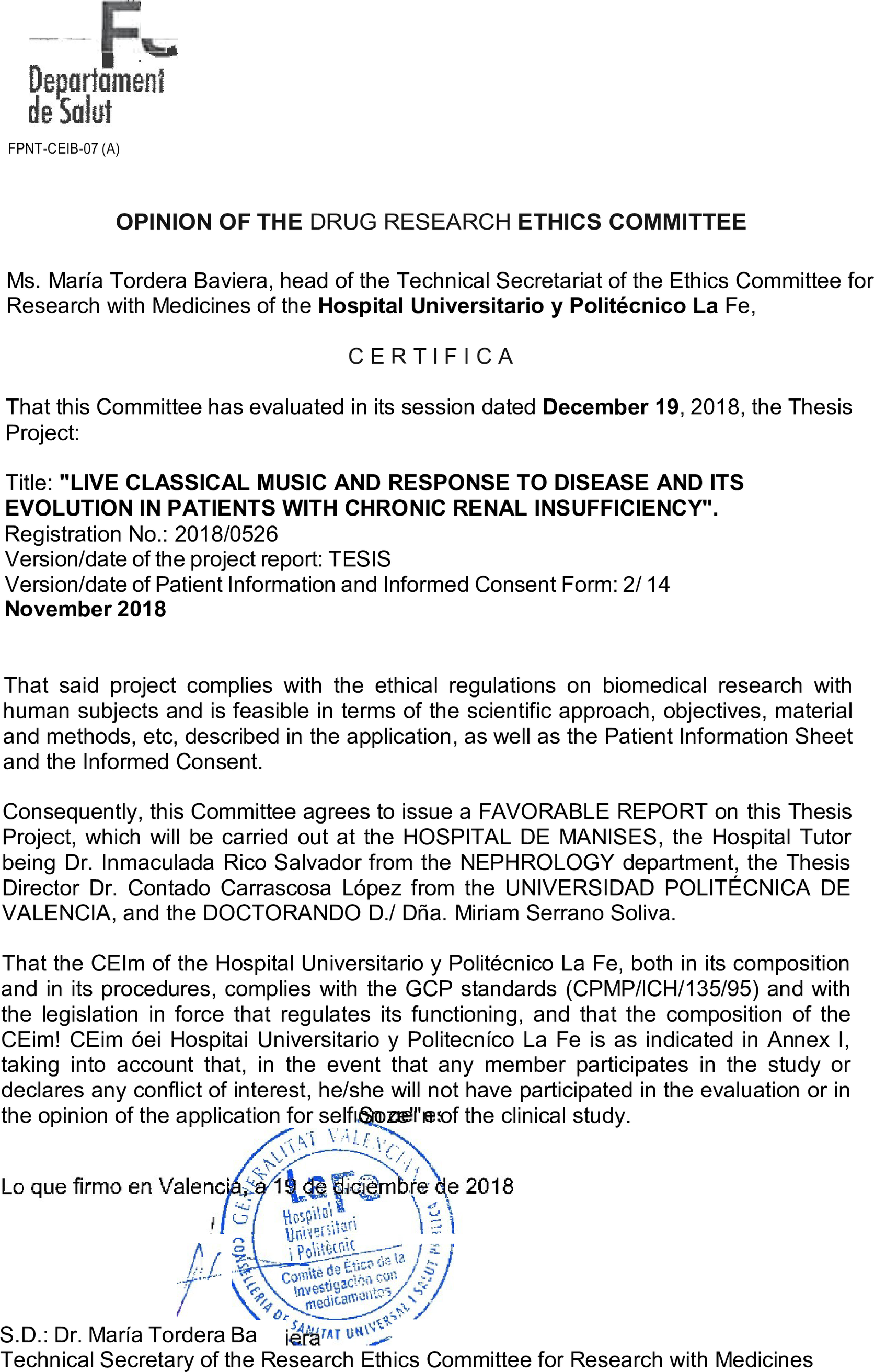

Supplement: S3 File — (TIF) [file pone.0307661.s003.tif]

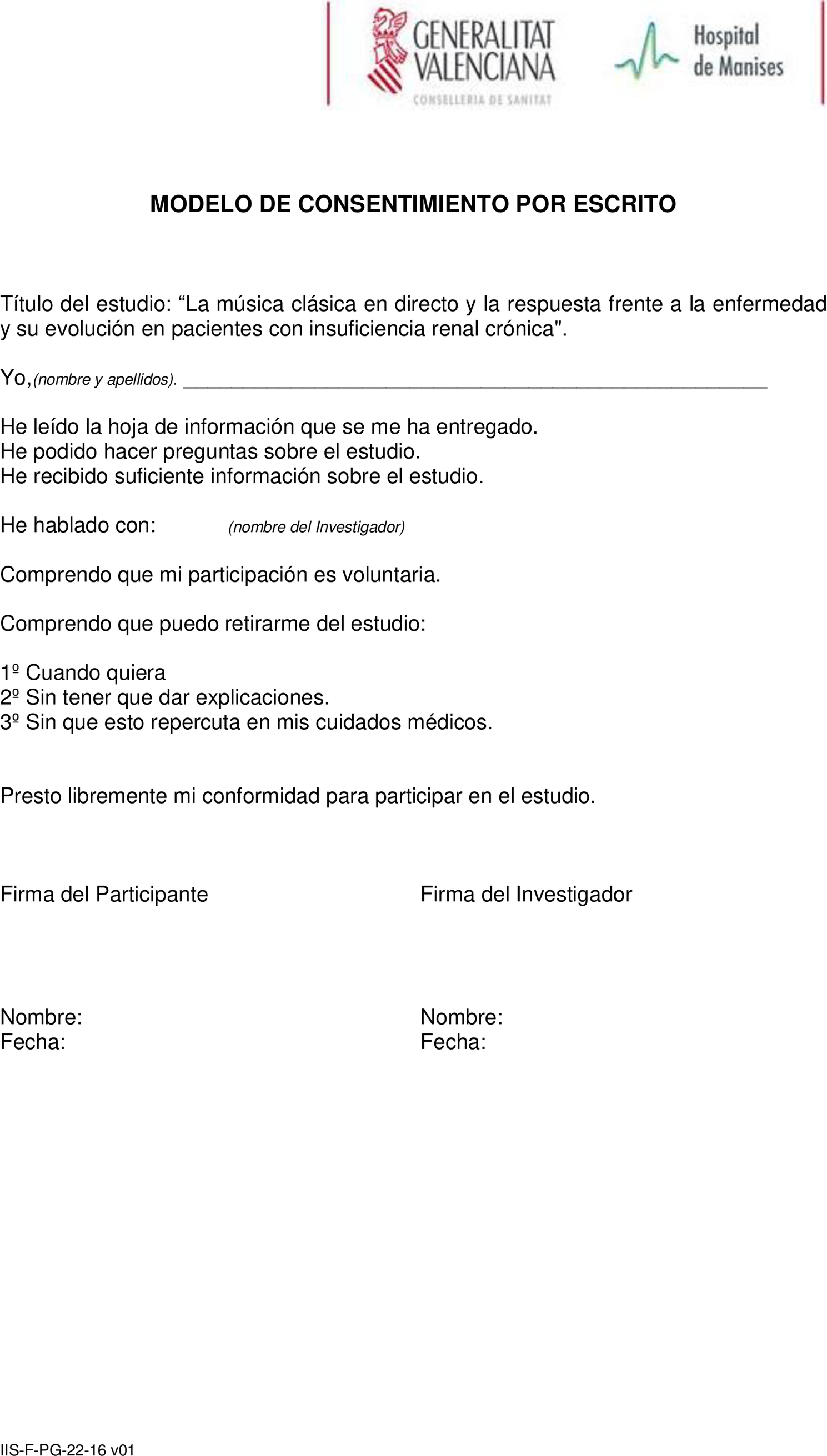

Supplement: S4 File — (TIF) [file pone.0307661.s004.tif]

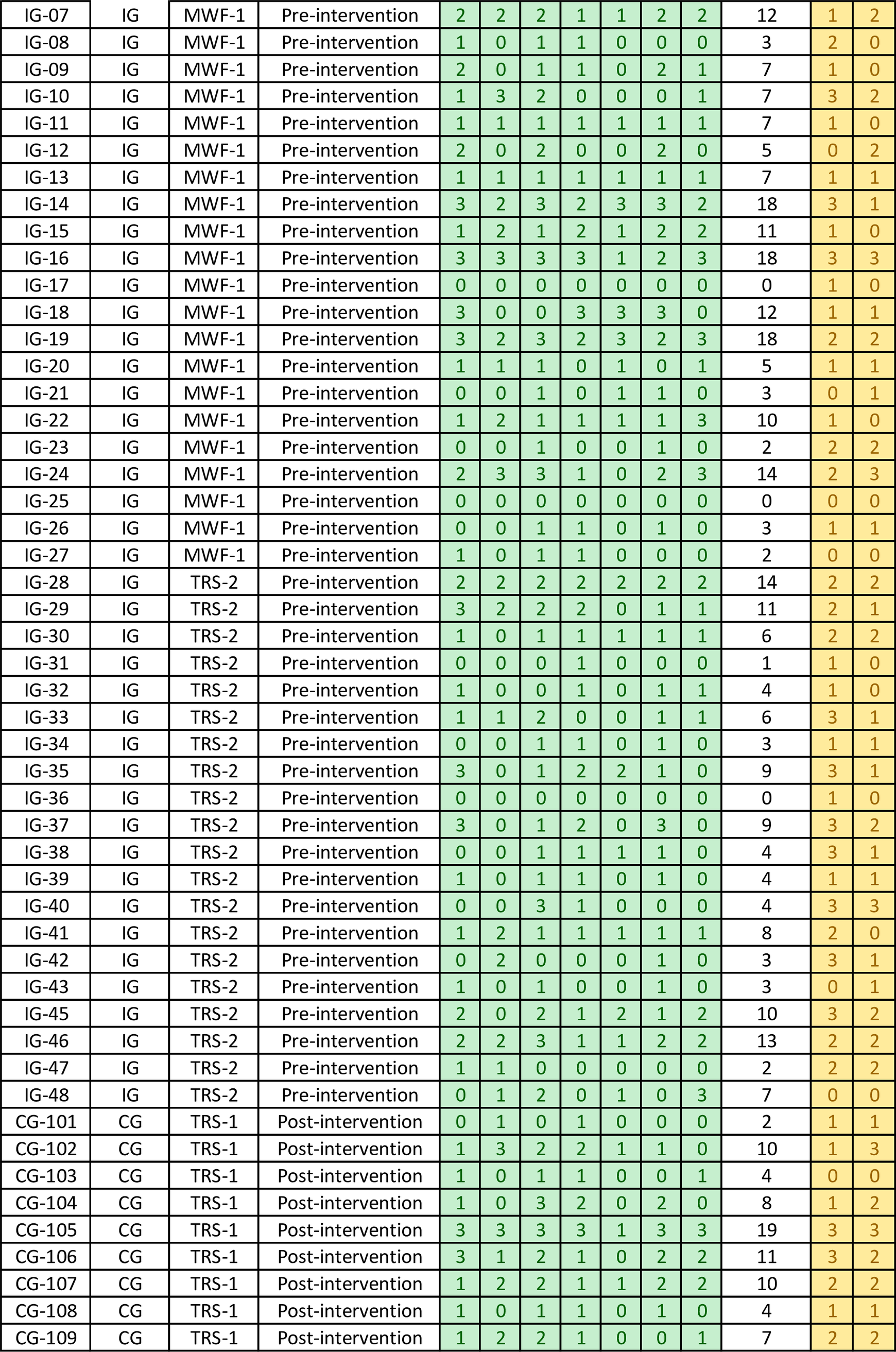

Supplement: S5 File — (ZIP) [file pone.0307661.s005.zip › S5.Supporting Information Data (10).tif]

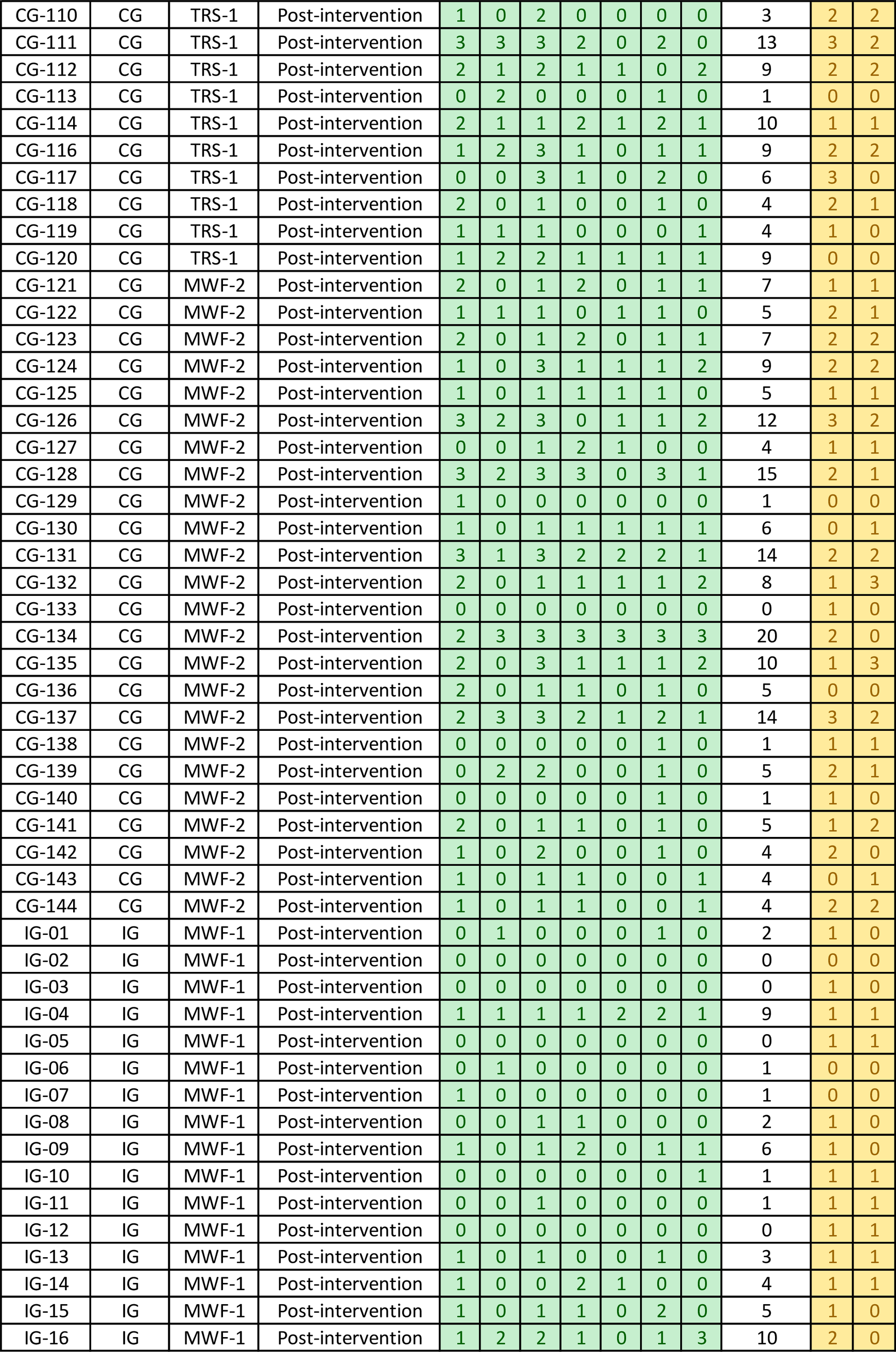

Supplement: S5 File — (ZIP) [file pone.0307661.s005.zip › S5.Supporting Information Data (11).tif]

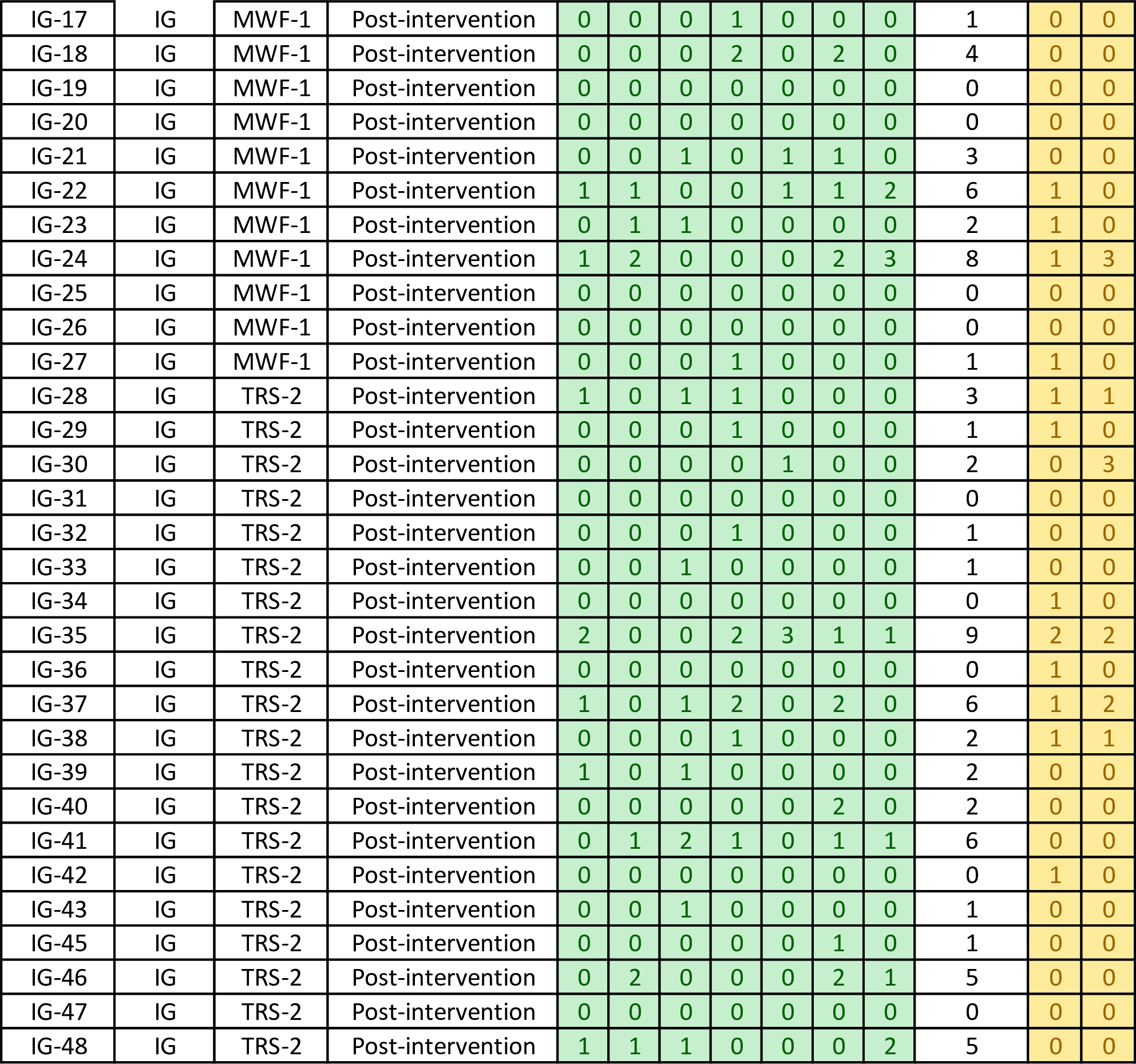

Supplement: S5 File — (ZIP) [file pone.0307661.s005.zip › S5.Supporting Information Data (12).tif]

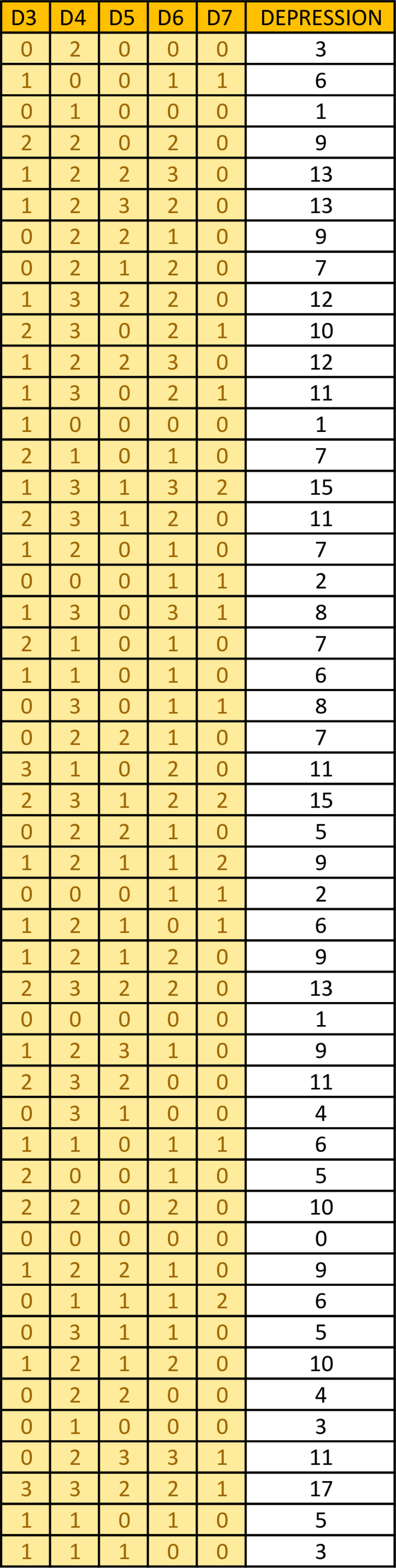

Supplement: S5 File — (ZIP) [file pone.0307661.s005.zip › S5.Supporting Information Data (13).tif]

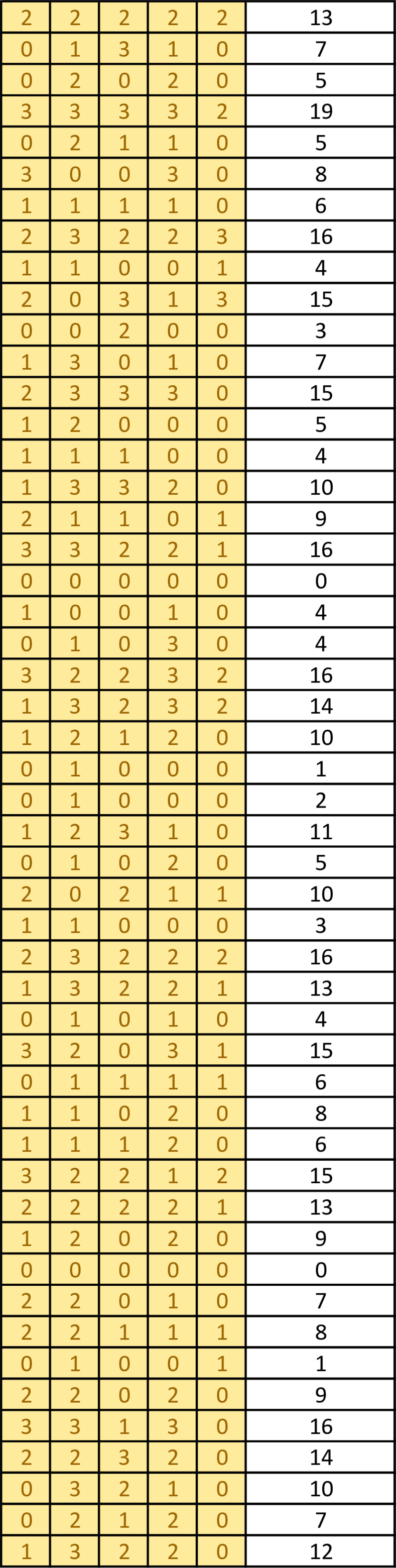

Supplement: S5 File — (ZIP) [file pone.0307661.s005.zip › S5.Supporting Information Data (14).tif]

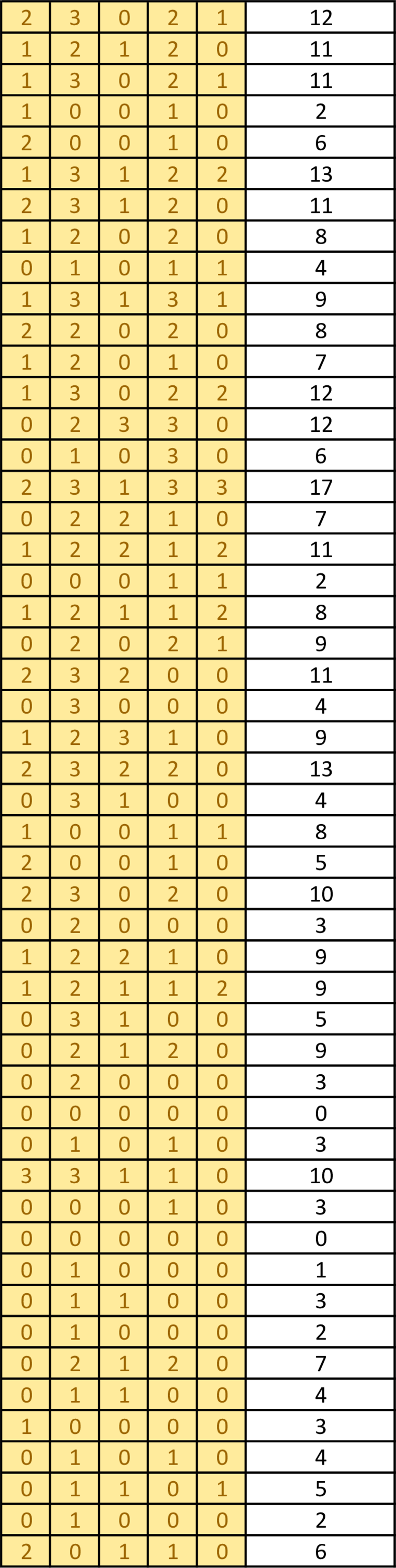

Supplement: S5 File — (ZIP) [file pone.0307661.s005.zip › S5.Supporting Information Data (15).tif]

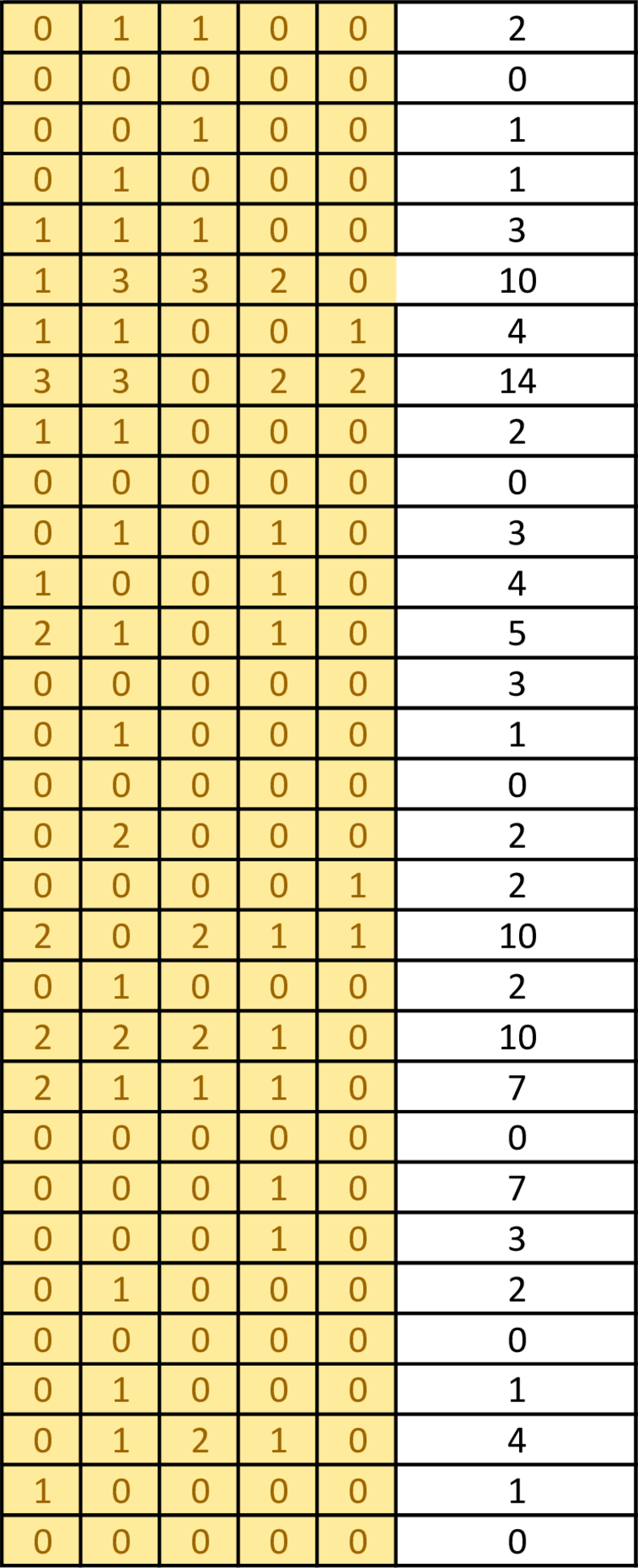

Supplement: S5 File — (ZIP) [file pone.0307661.s005.zip › S5.Supporting Information Data (16).tif]

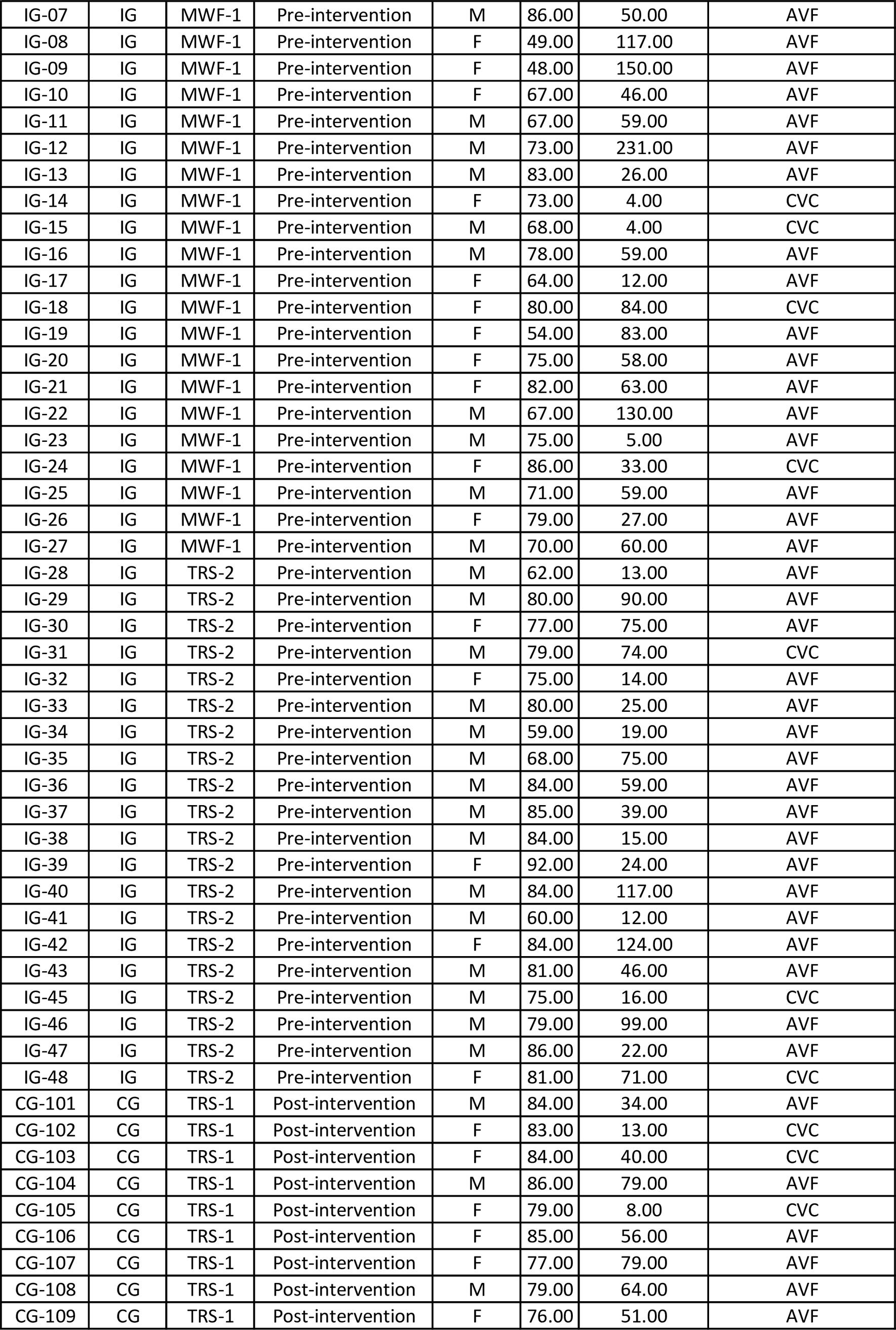

Supplement: S5 File — (ZIP) [file pone.0307661.s005.zip › S5.Supporting Information Data (2).tif]

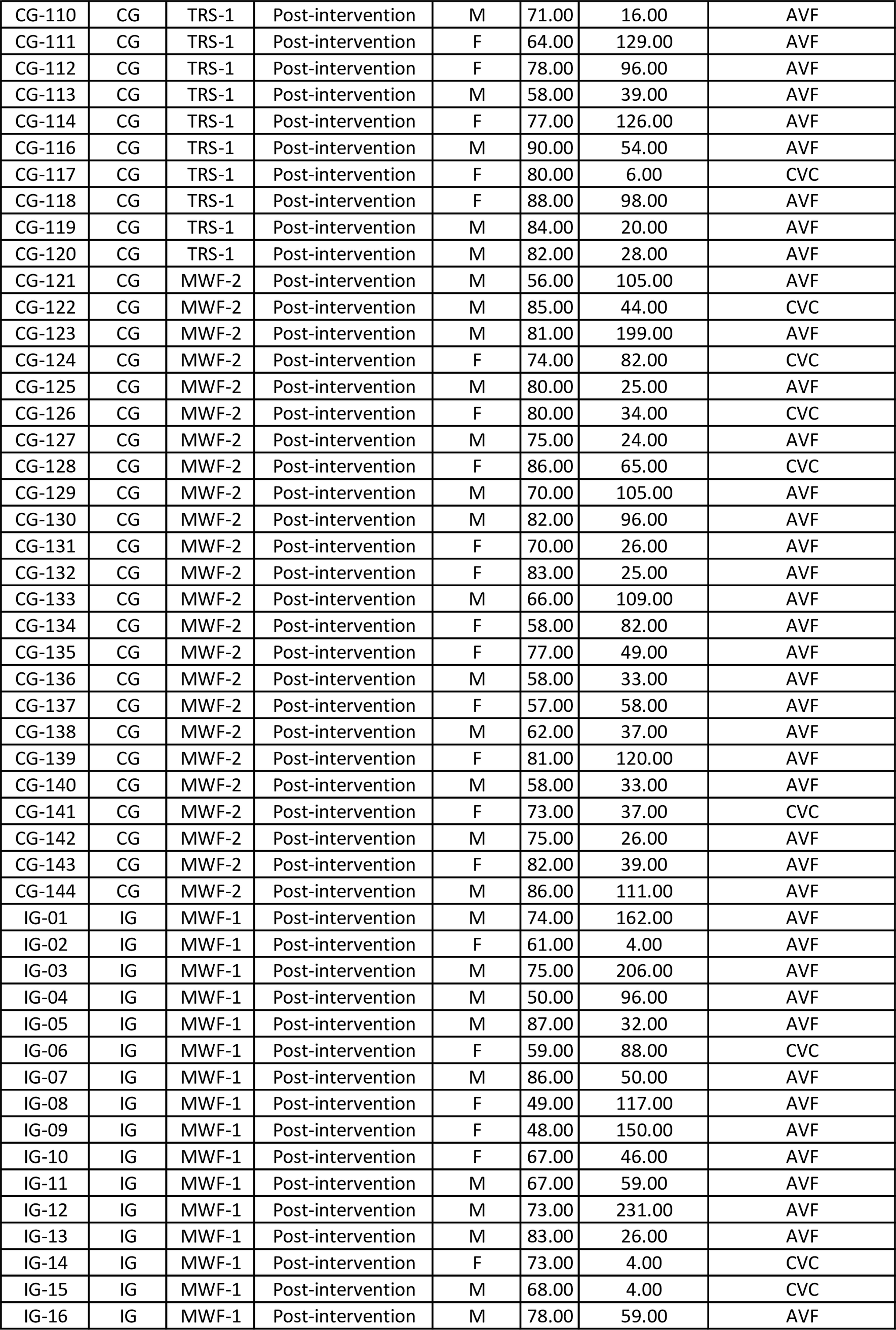

Supplement: S5 File — (ZIP) [file pone.0307661.s005.zip › S5.Supporting Information Data (3).tif]

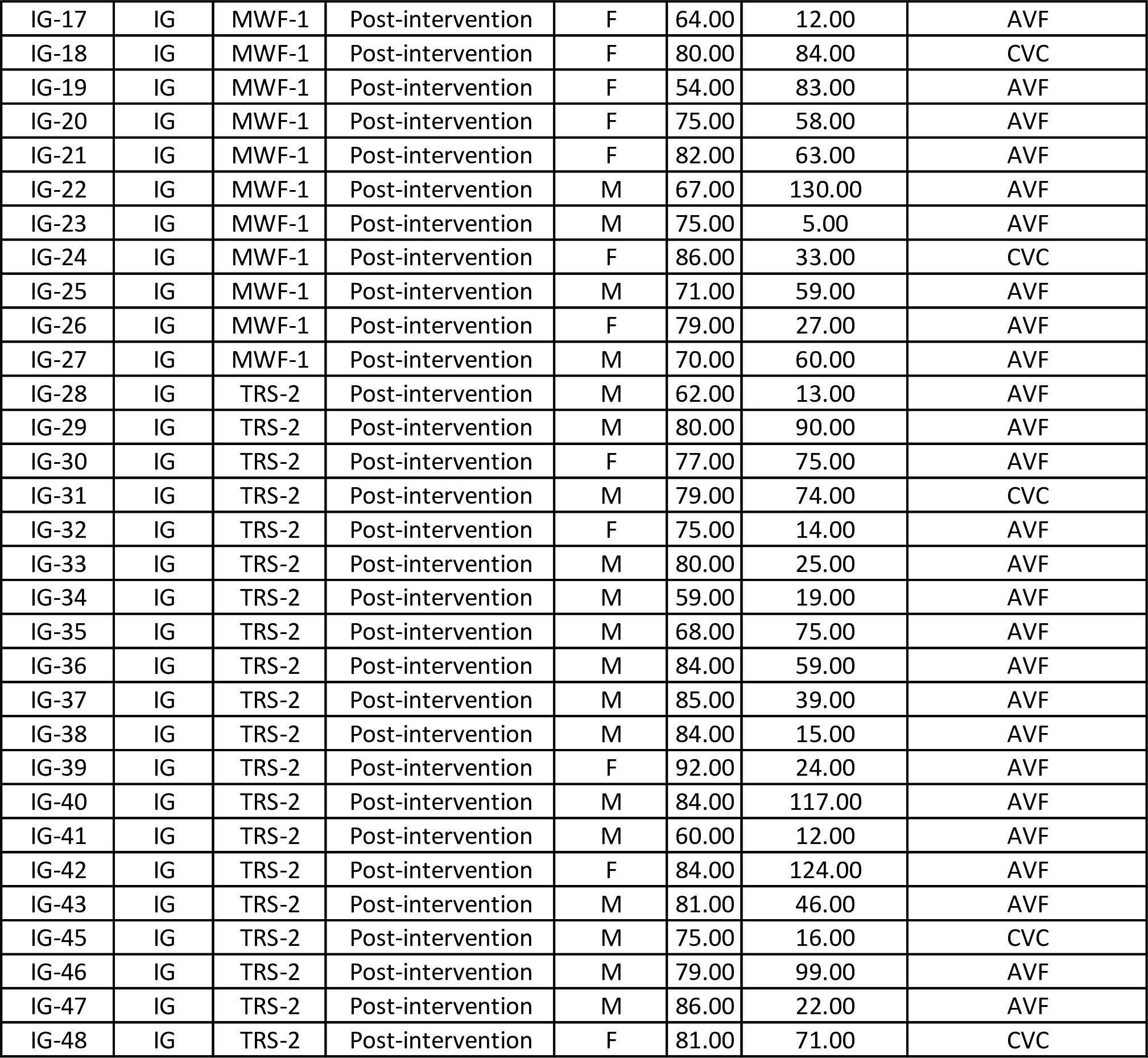

Supplement: S5 File — (ZIP) [file pone.0307661.s005.zip › S5.Supporting Information Data (4).tif]

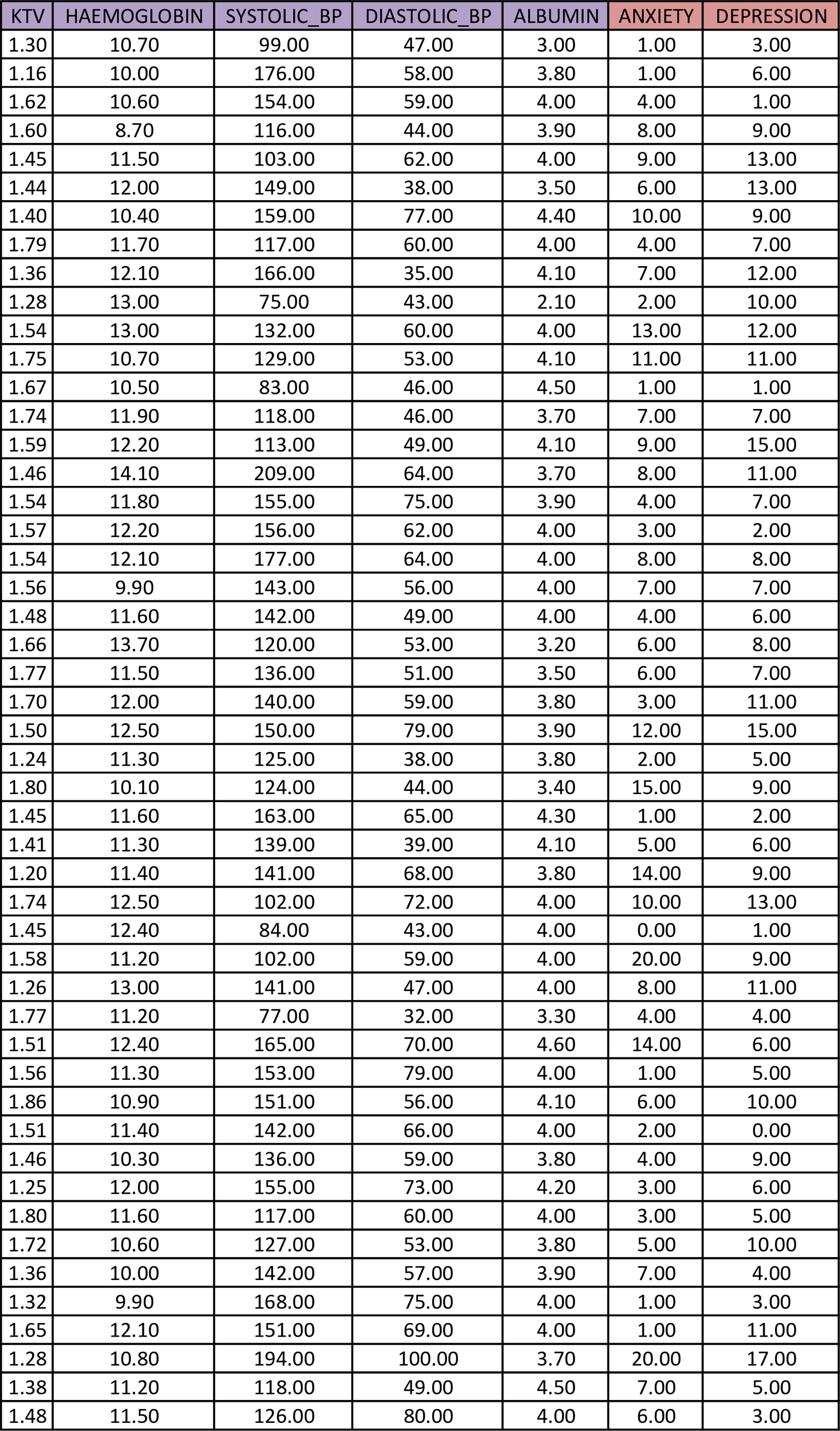

Supplement: S5 File — (ZIP) [file pone.0307661.s005.zip › S5.Supporting Information Data (5).tif]

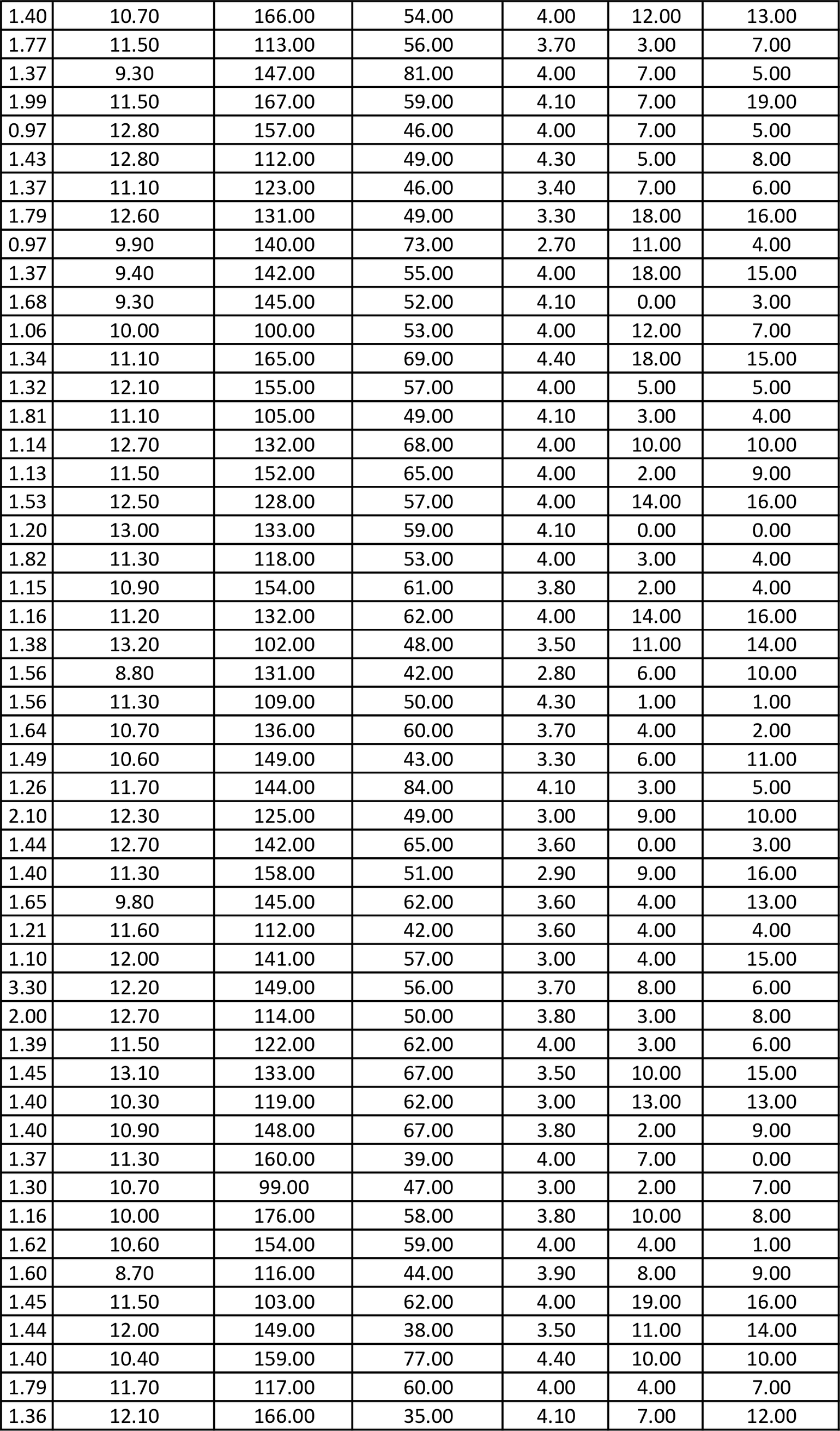

Supplement: S5 File — (ZIP) [file pone.0307661.s005.zip › S5.Supporting Information Data (6).tif]

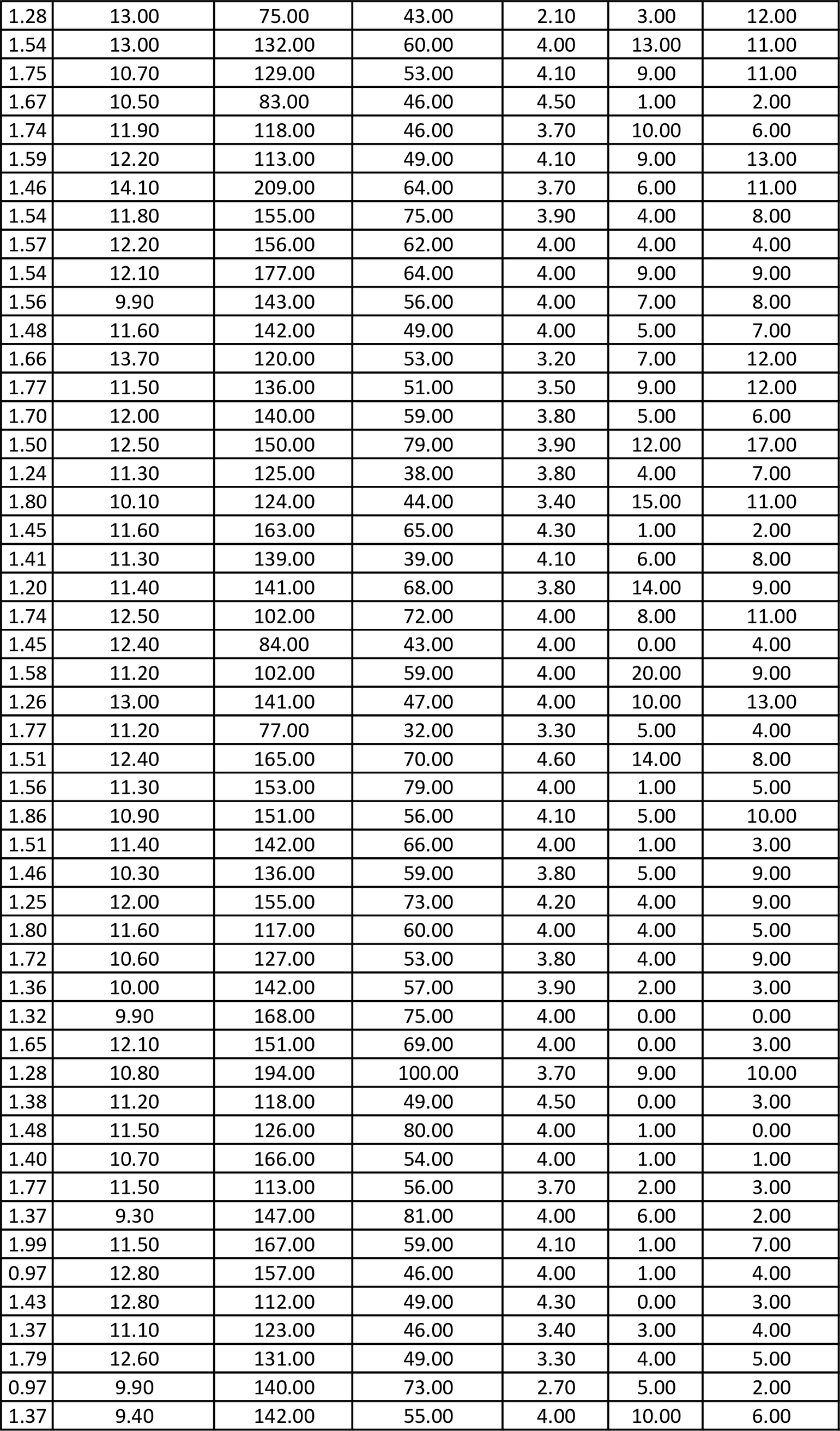

Supplement: S5 File — (ZIP) [file pone.0307661.s005.zip › S5.Supporting Information Data (7).tif]

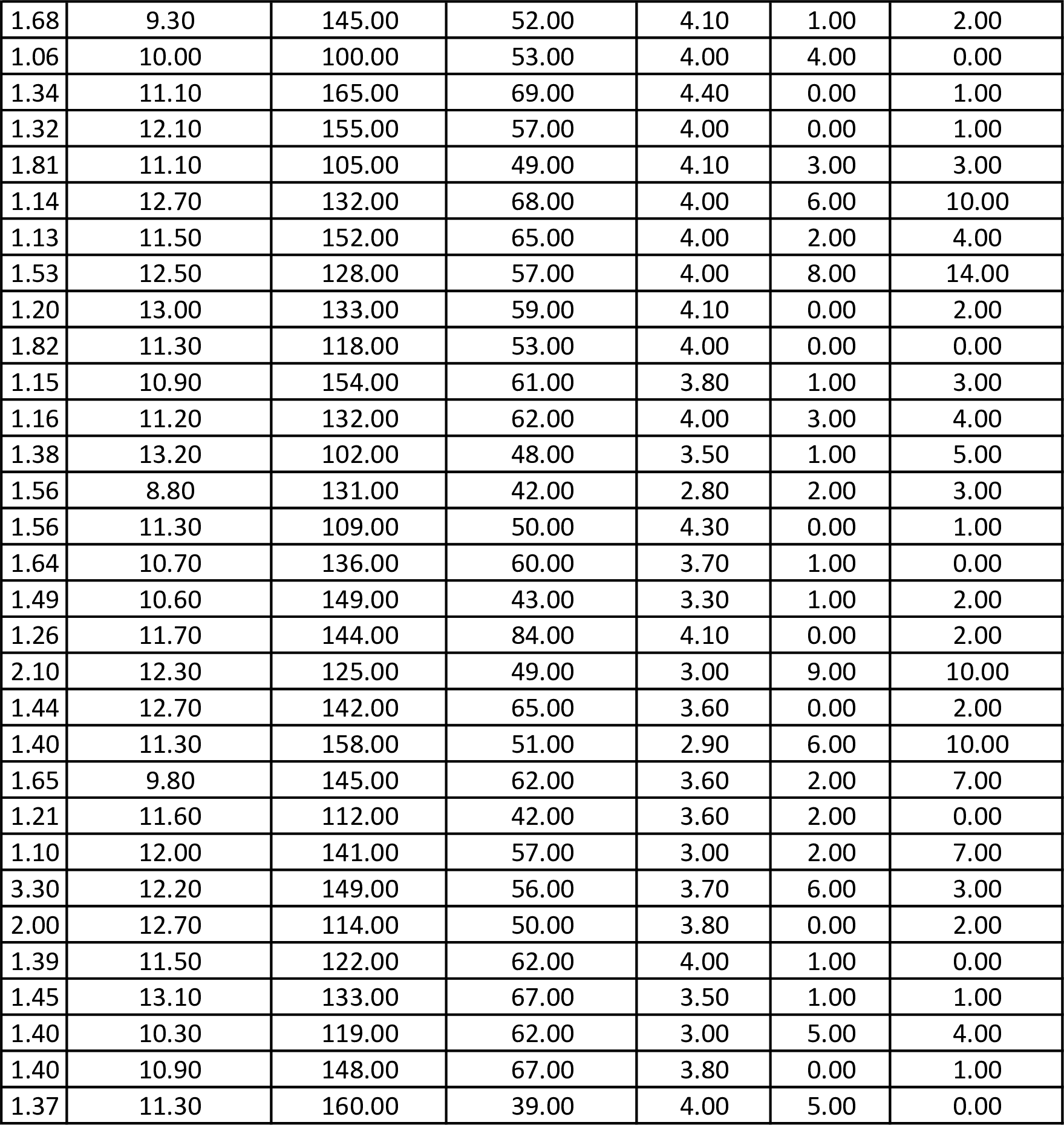

Supplement: S5 File — (ZIP) [file pone.0307661.s005.zip › S5.Supporting Information Data (8).tif]

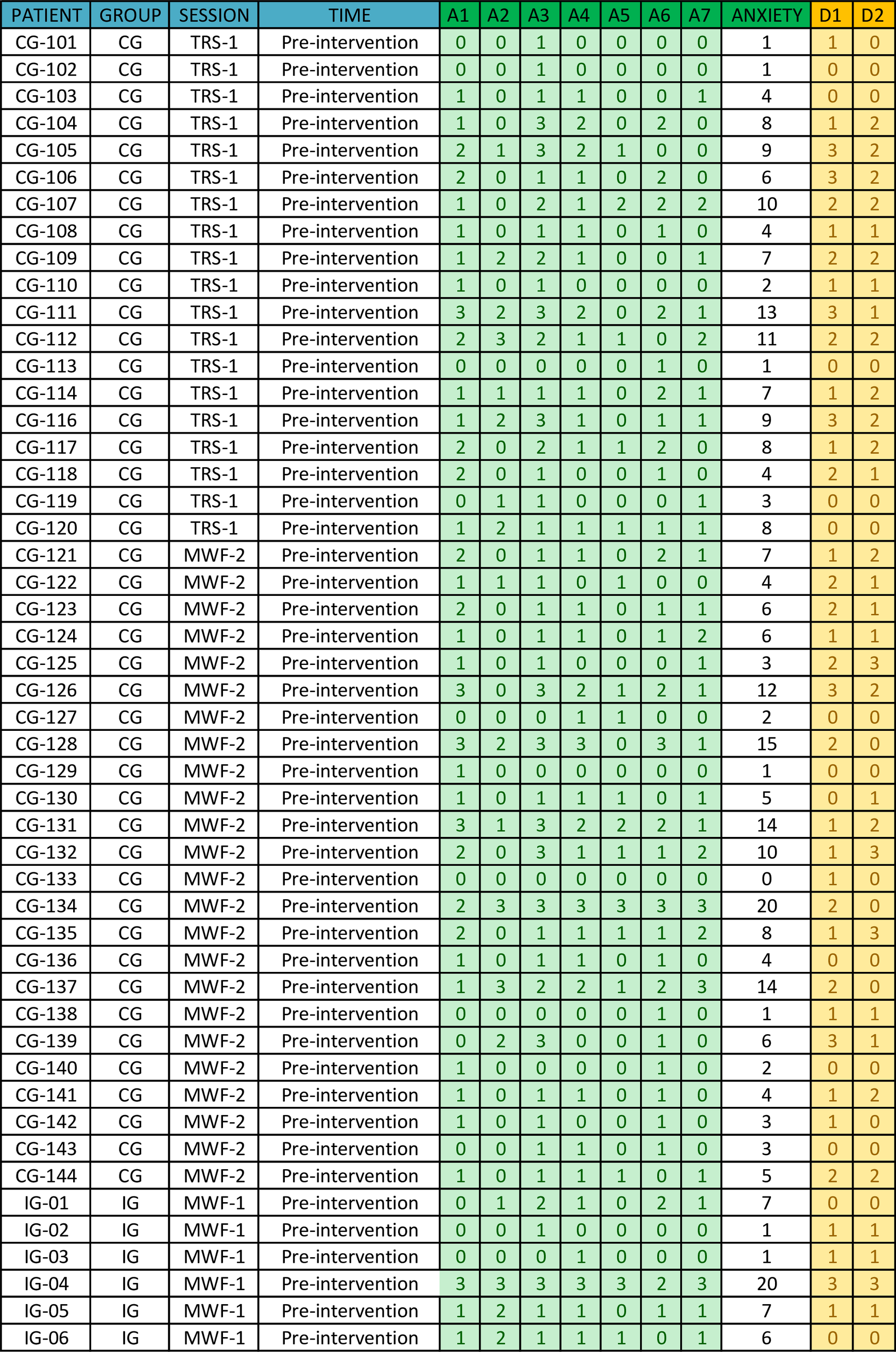

Supplement: S5 File — (ZIP) [file pone.0307661.s005.zip › S5.Supporting Information Data (9).tif]

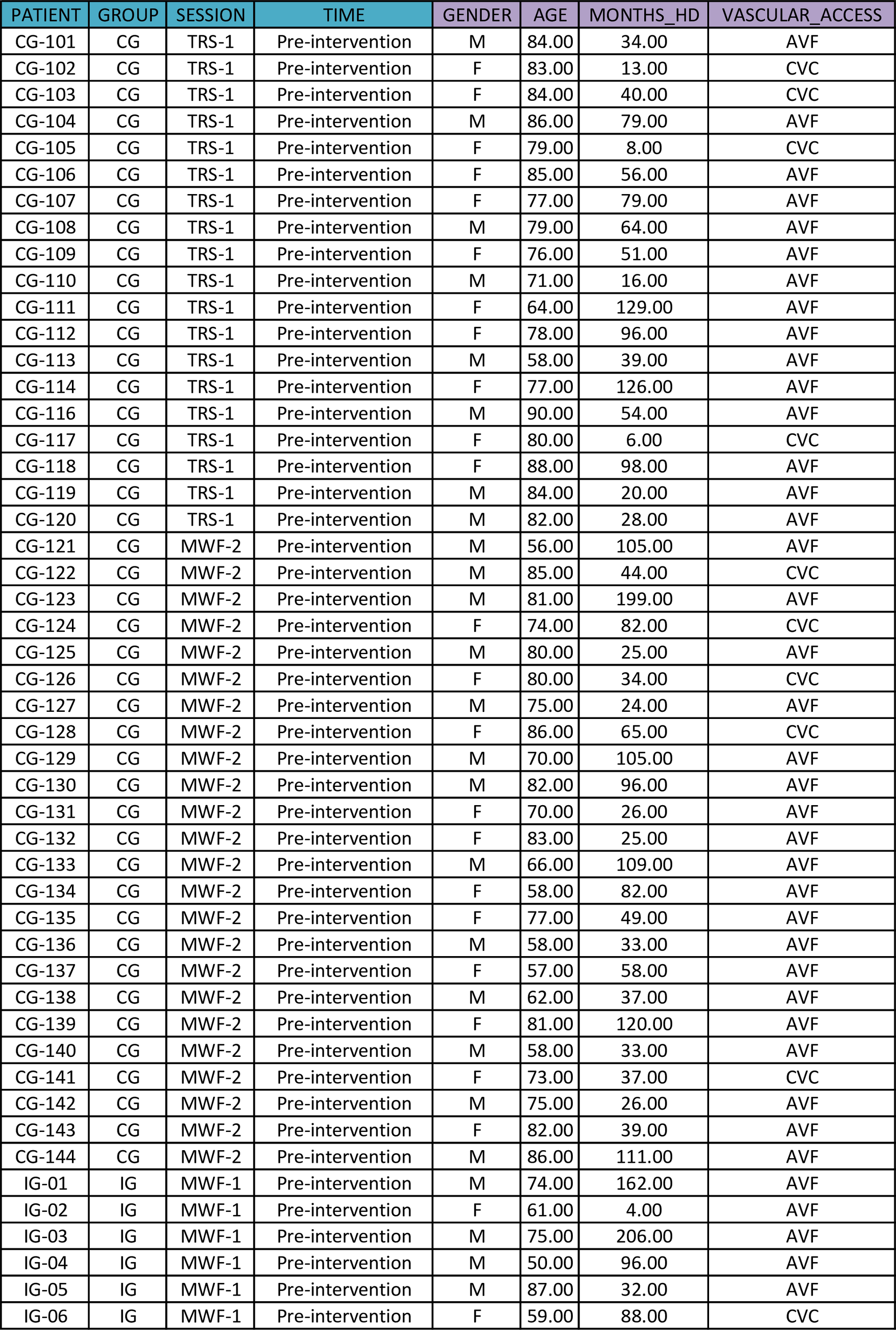

Supplement: S5 File — (ZIP) [file pone.0307661.s005.zip › S5.Supporting Information Data.tif]
